# Different responses to risperidone treatment in Schizophrenia: a multicenter genome-wide association and whole exome sequencing joint study

**DOI:** 10.1038/s41398-022-01942-w

**Published:** 2022-04-28

**Authors:** Mingzhe Zhao, Jingsong Ma, Mo Li, Wenli Zhu, Wei Zhou, Lu Shen, Hao Wu, Na Zhang, Shaochang Wu, Chunpeng Fu, Xianxi Li, Ke Yang, Tiancheng Tang, Ruoxi Shen, Lin He, Cong Huai, Shengying Qin

**Affiliations:** 1 Bio-X Institutes, Key Laboratory for the Genetics of Developmental and Neuropsychiatric Disorders (Ministry of Education), Shanghai Jiao Tong University, Shanghai, 200030 China; 2 School of Life Sciences and Biotechnology, Shanghai Jiao Tong University, Shanghai, 200240 China; 3 School of Engineering, Westlake University, 18 Shilongshan Road, Hangzhou, 310024 Zhejiang Province China; 4 Institute of Advanced Technology, Westlake Institute for Advanced Study, 18 Shilongshan Road, Hangzhou, 310024 Zhejiang Province China; 5 The Fourth People’s Hospital of Wuhu, No.1 East Wuxiashan Road, Wuhu, 241003 China; 6 The Second People’s Hospital of Lishui, No.69 Beihua Road, Lishui, 323020 China; 7 The Third People’s Hospital of Shangrao, No.1 Fenghuang East Avenue, Taokan Road, Shangrao, 334000 China; 8 Shanghai Yangpu district mental health center, No.585 Jungong Road, Yangpu District, Shanghai, 900093 China

**Keywords:** Schizophrenia, Clinical genetics

## Abstract

Risperidone is routinely used in the clinical management of schizophrenia, but the treatment response is highly variable among different patients. The genetic underpinnings of the treatment response are not well understood. We performed a pharmacogenomic study of the treatment response to risperidone in patients with schizophrenia by using a SNP microarray -based genome-wide association study (GWAS) and whole exome sequencing (WES)-based GWAS. DNA samples were collected from 189 patients for the GWAS and from 222 patients for the WES after quality control in multiple centers of China. Antipsychotic response phenotypes of patients who received eight weeks of risperidone treatment were quantified with percentage change on the Positive and Negative Syndrome Scale (PANSS). The GWAS revealed a significant association between several SNPs and treatment response, such as three *GRM7* SNPs (rs141134664, rs57521140, and rs73809055). Gene-based analysis in WES revealed 13 genes that were associated with antipsychotic response, such as *GPR12* and *MAP2K3*. We did not identify shared loci or genes between GWAS and WES, but association signals tended to cluster into the GPCR gene family and GPCR signaling pathway, which may play an important role in the treatment response etiology. This study may provide a research paradigm for pharmacogenomic research, and these data provide a promising illustration of our potential to identify genetic variants underlying antipsychotic responses and may ultimately facilitate precision medicine in schizophrenia.

## Introduction

Schizophrenia is a severe mental disorder with a heterogeneous combination of symptoms. Characteristic symptoms of schizophrenia can be divided into positive symptoms such as delusions and hallucinations, negative symptoms consisting of social withdrawal and affective flattening, as well as cognitive symptoms expressing as a broad set of cognitive dysfunctions. The average lifetime prevalence of schizophrenia is approximately 1% [1]. However, prevalence rates vary geographically by up to fivefold [2]. Schizophrenia accounts for significant health care costs, and is associated with a reduced life expectancy of approximately 15 years on average [3]. Antipsychotic medications are routinely used in the clinical management of schizophrenia, but the efficacy of antipsychotics in alleviating psychotic symptoms with response rates ranging from 66% for first-episode patients to 47% for chronic patients [4]. Patients on antipsychotics often experience a lengthy “trial-and-error” process marked by poorly managed symptoms before the optimal medications and doses are found. Consequently, one-year discontinuation rates may be as high as 74% due to lack of efficacy and tolerability [5]. In addition, this “trail-and-error” approach also creates tremendous social and economic burdens in various ways, as unsuccessful treatments ultimately lead to waste of medical resources and can be a risk to public safety [6, 7]. Therefore, there is a critical need to discover effective predictors of drug efficacy, or patients will continue to suffer unnecessarily.

It is generally believed that understanding the genetic determinants of drug response will help to guide therapeutic strategies toward a better efficacy profile [8]. Pharmacogenetics is the field of research and clinical practice that focuses on the influence of genetics on drug response. Primary pharmacogenetic studies based on prior knowledge have investigated several candidate genes that are suggested to be considered when prescribing medications used in psychiatry. Commonly used candidates in pharmacogenetics include genes involved in the absorption, distribution, metabolism or excretion of drugs (e.g., *CYP2D6*, *CYP2C19*, *CYP2C9*, *ABCB1*, *SLC6A4*, *COMT*) or genes involved in the immune system (e.g., *HLA-A*, and *HLA-B)* [8]. However, candidate gene approaches mostly focus on potential genes with functional relevance, and there can still be unknown genes or unidentified genetic variants that may help determine the drug response. The whole-genome application of pharmacogenetics termed pharmacogenomics enables contributions from novel and less obvious genes to be detected, especially in the area of drug-target genetics, which is more complex and less well understood than the pharmacogenetics of drug metabolism [9]. Yu et al. conducted a large pharmacogenomic genome-wide association study (GWAS) using the Chinese Antipsychotics Pharmacogenomics Consortium (CAPOC) and the Chinese Antipsychotics Pharmacogenetics Consortium (CAPEC) samples and found that single-nucleotide polymorphisms (SNPs) in *MEGF10*, *SLC1A1*, and *PCDH7* were associated with antipsychotic treatment response [10]. Overall, 20.8% of the total variation in response to antipsychotics was attributed to common SNPs across the genome [10]. However, almost without exception, even this well-powered GWAS identified loci that could explain only a small proportion of the genetic variance for antipsychotic treatment response, revealing the so-called missing heritability problem [11, 12]. Incomplete linkage disequilibrium between the causal variants and common SNP markers may explain a small part of the heritability underestimation. More importantly, a large number of rare variants with large effects were missed by current GWAS [13, 14]. Whole-exome sequencing (WES) offers a cost-effective strategy for investigating rare variants in drug-response studies [15]. Wang et al. conducted a pharmacogenomic WES study which found a greater burden of rare damaging variants in the reduced NMDA(*N*-methyl-D-aspartate)-mediated synaptic currents and reduced AMPA (α-amino-3-hydroxy-5-methy-4-isoxazole propionic acid)-mediated synaptic current gene sets curated in patients with poor response to antipsychotic medications, but no SNPs and single gene achieved genome-wide significance [16]. It is worth noting that most of the large sample GWAS and WES studies mainly used samples treated with different antipsychotics that have varying pharmacokinetic properties and mechanisms of action. Mixed antipsychotics for pharmacogenomic study may lead to spurious associations that often cannot be replicated due to heterogeneity [17].

The common variants are thought to impart subtle effects on gene function, often through changes to gene regulation [18]. The rare variants may have larger effects on gene function, such as nonsynonymous variants that alter the amino acid sequence of the resulting protein, and as a result lead to large changes in drug response. Multiple variants contributing to drug response are segregating in the population at a wide allele frequency spectrum, and a combination of common and rare variants may be required to obtain a more complete picture of the genetic architecture of drug response [19].

To investigate the genetic mechanisms underlying the difference in treatment response, we performed a discovery GWAS and WES to analyze the role of both common and rare variants of response to eight weeks of acute-phase treatment with risperidone and contribute to the development of personalized antipsychotic prescriptions.

## Materials and methods

### Study participants

This study was conducted in accordance with the Strengthening the Reporting Of Pharmacogenetic Studies (STROPS) reporting guideline [20]. Subjects were recruited from the inpatient departments of psychiatric hospitals in Shanghai, Wuhu, Lishui, and Shangrao, China. The inclusion criteria for participants were as follows: Han Chinese ancestry, diagnosis of schizophrenia by two psychiatric physicians without divergence based on the Structured Clinical Interview of DSM-IV, total Positive and Negative Syndrome Scale (PANSS) scores of more than 60, physically healthy with all laboratory parameters within normal limits, taken oral medication and written informed consent. Participants were excluded from the study if they had severe or unstable physical disease; were pregnant or breastfeeding; were diagnosed with schizoaffective disorder, schizophreniform disorder, delusional disorder, or other cognitive disorders; did not have a guardian.

This study was approved by the Ethical Committee of Bio-X Institutes of Shanghai Jiao Tong University. All participants were asked to appoint a legal guardian to provide written informed consent and help patients with decision making before enrolling in this study.

### Phenotyping

The treatment response was evaluated by percentage change on PANSS to antipsychotic medication. During an eight weeks of risperidone treatment, drug efficacy was evaluated four times (baseline, week two, week four, week eight) based on the change in PANSS scores from baseline. The PANSS reduction rate for each participant at week eight was calculated by the following formula. The 50% was set as threshold to define responders (PANSS reduction rate ≥ 50%) and nonresponders (PANSS reduction rate < 50%).

$${{{\mathrm{PANSS}}}}\;{{{\mathrm{percentage}}}}\;{{{\mathrm{change}}}} = \frac{{{{{\mathrm{PANSS}}}}\;{{{\mathrm{week}}}}\;{{{\mathrm{eight}}}}\;{{{\mathrm{scores}}}} - {{{\mathrm{PANSS}}}}\;{{{\mathrm{baseline}}}}\;{{{\mathrm{scores}}}}}}{{{{{\mathrm{PANSS}}}}\;{{{\mathrm{baseline}}}}\;{{{\mathrm{scores}}}}}} \times 100$$

### Chip genotyping

Genomic DNA was extracted from peripheral blood with the QIAamp DNA Blood Mini Kit (QIAGEN GmbH, Hilden, Germany) and quality control (QC) assessment for purity, degradation after collection, storage, and extraction was conducted by the recommended manufacturers. Initially, 196 patients were genotyped with the Illumina Global Screening Array-24 v1.0 Beadchip (Illumina, San Diego, CA, USA). Samples and markers underwent QC before the association analysis using PLINK software (version 1.9, http://www.cog-genomics.org/plink). Samples were excluded if the genotype call rate was less than 95% and the heterozygosity rate was greater than three sigma, if they were genetic outliers, or if they had blood relationship. SNPs with minor allele frequency less than 0.01, the genotype call rate less than 95%, and Hardy-Weinberg equilibrium *P* values less than 1 × 10^−6^ were removed. Principal component analyses (PCAs) were conducted to identify genetic outliers. Genotype imputation was conducted with the prephasing imputation stepwise approach implemented in SHAPEIT and IMPUTE2 [21, 22], and the reference haplotypes were derived from phase I of the 1000 Genomes Project (release version 3). SNPs with imputation quality scores less than 0.8 were removed from further analyses.

### Whole exome DNA sequencing

There were 227 individuals being designed for the WES. Whole-exome capture libraries were constructed with Agilent SureSelect Human All Exon V6 (Agilent Technologies, Santa Clara, CA, USA) following the manufacture’s protocols and then were assessed with Agilent 2100 Bioanalyzer High Sensitivity DNA chip (Agilent Technologies, Santa Clara, CA, USA). Trusted high-level libraries were sequenced on Illumina X10 (Illumina, San Diego, CA, USA).

Raw sequencing reads with FASTQ format were mapped to the human reference genome (hg19) using the Burrows-Wheeler Aligner tool (BWA, version 0.7.17, http://bio-bwa.sourceforge.net/bwa.shtml). Polymerase chain reaction (PCR) duplicates were detected with the Picard tool (version 2.15.0, http://broadinstitute.github.io/picard/) using the mapped reads. Then, the Genome Analysis Toolkit (GATK, version 3.8, https://software.broadinstitute.org/gatk/) was applied for local realignment and quality score recalibration (BQSR), which can realign insertion-deletions (indels) and correct for the base quality scores from BAM files. The HaplotypeCaller tool embedded in GATK was applied to call single nucleotide variants (SNVs). Every called variant file with VCF formats was merged using the GenotypeGVCFs tool and then filtered using the VariantRecalibrator tool embedded in GATK.

### Common SNP association analysis

Association analyses between genetic variants, age, sex, and the first five principal components of population structure and PANSS percentage change values were assessed with linear regression under an additive genetic model implemented in PLINK (version 1.9 beta). Given differences in sample size, phenotypic characterization, and effect size between pharmacogenomic GWAS and complex-disease GWAS [23], an accepted genome-wide significance threshold on the order of *P* < 1 × 10^−5^ was used for our pharmacogenomic GWAS [24]. A *P* value less than 5 × 10^−5^ was reported as a finding of interest because markers associated with important individual differences in treatment response could be moderately significant.

Several secondary analyses were conducted based on the results of GWAS. The tissue-specific expression patterns of genes in human tissues in genotype-tissue expression Portal (GTEx, http://www.gtexportal.org/home/) were investigated. The GTEx database collected approximately 17382 RNA-seq samples across 54 tissues from 948 postmortem donors [25]. We explored expression quantitative trait loci (QTL) data in the brain eQTL database, the Brain Expression Consortium (BRAINEAC) (http://caprica.genetics.kcl.ac.uk/BRAINEAC/). The BRAINEAC database collected 134 neuropathologically normal donors from the MRC Sudden Death Brain Bank in Edinburgh and Sun Health Research Institute. The eQTL data could be generated for ten human brain regions in the BRAINEAC database and a mean expression profile was also calculated across the ten brain regions to find weaker but ubiquitous signals in the human brain [26].

### Rare SNV association analysis

For rare SNV testing of WES, gene-based association analysis was performed to analyze the combined effects of coding region SNVs (including both synonymous and nonsynonymous SNVs or including only nonsynonymous SNVs) with a minor allele frequency lower than 0.01 of the East Asian population in public databases, such as the 1000 Genomes Project, ExAC and gnomAD-WES datasets. The gene-based association analysis was assessed using three algorithms (CMC, PRICE, SKAT-O) in RVTESTS [27, 28].

## Results

### GWAS

We totally recruited 226 patients with schizophrenia in multiple centers of China (Shanghai:102, Wuhu:39, Lishui:32, Shangrao:53) and divided them into two groups (responder group/nonresponder group, Shanghai:28/74, Wuhu:8/31, Lishui:8/24, Shangrao:22/31) based on the percentage change in PANSS after an eight weeks of risperidone treatment. The total response rate is 29.2% under the treatment of risperidone in our samples. Significant differences were found between the two groups in baseline PANSS total scores, endpoint PANSS total scores, and PANSS percentage changes but not on age and sex (Table 1). Among 196 genotyped patients, 189 of them remained after quality control. All patients included in the GWAS were unrelated Han Chinese without population stratification after principal component analysis. There are 700,078 genotyped SNPs in raw chip data. After imputation and quality control, a linear regression analysis with 446,504 common SNPs on the PANSS percentage change values was conducted.

**Table 1:** Demographic and clinical characteristics of samples.

Variables	Responders Group	Nonresponders Group	t/X^2^	P value
Age, mean (SD)	36.40 (10.621)	38.48 (12.205)	−1.197	0.233
Sex				
Male, No. (%)	38 (57.6)	84 (52.5)	0.485	0.486
Female, No. (%)	28 (42.4)	76 (47.5)		
Clinical assessment, mean (SD)				
Baseline PANSS total scores	80.70 (16.423)	85.74 (14.466)	−2.295	0.023
Endpoint PANSS total scores	35.73 (4.056)	51.43 (9.856)	−12.509	0.000
PANSS percentage changes	89.749 (6.322)	61.81 (12.050)	17.853	0.000

*PANSS* positive and negative syndrome scale.

**Fig. 1:**
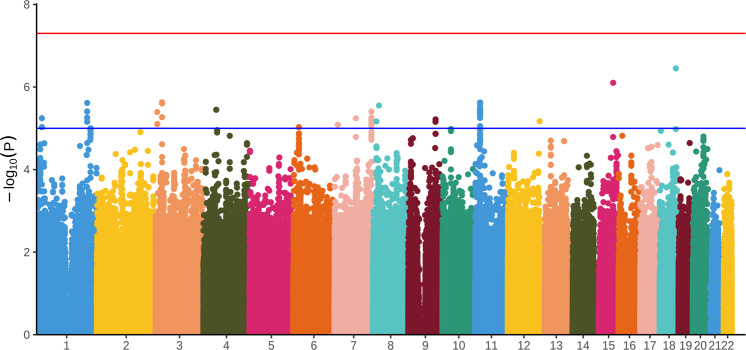
Manhattan plots of *P* values against their respective chromosomal positions for the genome-wide association study. The bule line corresponds to the *P* < 1 × 10^−5^, while the red line represents the genome-wide significance level (*P* < 5 × 10^−8^).

Figure 1 illustrates the Manhattan plot of the GWAS common SNP association results. As the Fig. 1 shows that 312 SNPs were weakly associated with risperidone treatment response (i.e., *P* < 5 × 10^-5^), most of which were intergenic variants that could not be annotated with gene symbols, and the nonintergenic variants with gene symbols were listed in Supplementary Table S1. Nevertheless, 185 SNPs showed genome-wide significant associations with treatment response to risperidone (i.e., *P* < 1 × 10^-5^), approximately half of which were located in the intergenic region, and the remaining SNPs were mainly located in *GRM7*, *PRMT3*, *USP48*, *SGCZ*, *DOCK5*, *HTATIP2*, and *PALM2AKAP2* (Supplementary Table S2).

The above seven mapped genes were then investigated for tissue-specific expression distribution using the GTEx database. Five genes (*GRM7*, *PRMT3*, *USP48*, *SGCZ*, *DOCK5*) were all highly expressed in brain tissue (Supplementary Fig. S1). We also conducted eQTL analysis in the BRAINEAC database. The results indicated that 11 SNPs in PRMT3 were associated with the mRNA expression of *HTATIP2*, *NAV2*, *NELL1*, *PRMT3*, and *SLC6A5*, and seven SNPs in *HTATIP2* were associated with the mRNA expression of *NAV2*, *SLC6A5*, and *PRMT3* in average-ten brain regions. The other SNPs were not associated with gene expression (Supplementary Table S3).

### WES

**Fig. 2:**
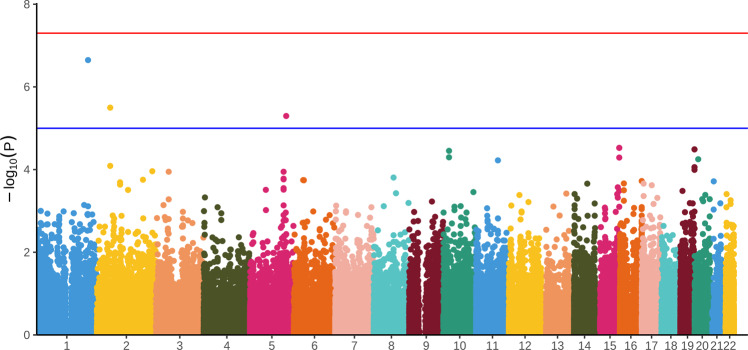
Manhattan plots of *P* values against their respective chromosomal positions for whole exome sequencing study. The bule line corresponds to the *P* < 1 × 10^−5^, while the red line represents the genome-wide significance level (*P* < 5 × 10^−8^).

The WES data were generated on the 226 patients and 222 of them remained after quality control. The data had an average per-target depth of coverage of × 74.57, with 97.5% of all targeted bases covered at × 10 or greater (94.1% at ≥20×). After taking a series of quality controls, 108,813 SNVs were identified in the protein-coding regions. Figure 2 depictes the Manhattan plots of the common SNP-based association analysis. Four SNPs showed a genome-wide significant association with the treatment response to risperidone, in *PACC1*, *SPTBN1*, and *HMGXB3* (i.e., *P* < 1 × 10^–5^), and seven SNPs were weakly associated with the risperidone treatment response (i.e., *P* < 5 × 10^–5^) (Table 2). For rare SNVs, we identified ten genes that reached the significance (*P* < 0.05) of all three algorithms (CMC, PRICE, SKAT-O) in RVTESTS in gene-based association analyses (Table 3).

**Table 2:** Genomic regions of common variants in the whole exome sequencing analyses.

CHR	SNP	Position	A1	A2	Gene	variant location	BETA	*P*
1	rs117462017	212587381	A	G	*PACC1*	synonymous variant	−22.78	2.25E–07
2	rs2271323	54871519	T	C	*SPTBN1*	synonymous variant	−34.24	3.18E–06
2	rs2271326	54864964	G	A	*SPTBN1*	intron variant	−34.24	3.18E–06
5	rs186497515	149410319	A	T	*HMGXB3*	missense variant	−30.33	5.05E–06
15	rs181987	99679655	A	C	*TTC23*	intron variant	−21.06	2.98E–05
15	rs260089	99678145	A	G	*TTC23*	3 prime UTR variant	−21.06	2.98E–05
15	rs7167599	99672599	C	G	*TTC23*	downstream gene variant	−21.06	2.98E–05
19	rs11673029	58491627	C	T	*ZNF606*	missense variant	−10.01	3.24E–05
19	rs9304808	58514183	C	T	*ZNF606*	5 prime UTR variant	−10.01	3.24E–05
19	rs9304809	58514475	T	G	*ZNF606*	5 prime UTR variant	−10.01	3.24E–05
10	rs149024826	23578843	C	T	*C10orf67*	missense variant	−22.08	3.52E–05

*CHR* chromosome, *SNP* single-nucleotide polymorphism, A1 minor allele, A2 major allele.

**Table 3:** Gene-based association results in the whole exome sequencing analyses.

Gene	P (CMC)	P (PRICE)	P (SKAT-O)
*MAP2K3*	3.004E–06	0.000	2.673E–06
*C20orf194*	2.553E–05	0.0002	0.0002
*CCDC188*	4.021E–05	0.0001	7.183E–05
*FAM182B*	0.0001	0.0003	0.0002
*FOXP1*	0.0003	0.002	0.0003
*NHLRC2*	0.0003	0.002	0.0003
*CDK11B*	0.0007	0.0007	0.0006
*GPR12*	0.0003	0.002	0.0003
*ATOH8*	0.003	0.003	0.003
*CNTLN*	0.002	0.004	0.002

*CMC* combined multivariate and collapsing, *SKAT-O* optimized sequence kernel association test.

### Convergent pathways between GWAS and WES

The results of the GWAS were correlated with those of the WES, which were represented in commonalities between genetic pathways identified through GWAS and WES. *GRM7* in the GWAS is the G-protein coupled receptor (GPCR) for glutamate, which is one of the most important neurotransmitters in the central nervous system. *GPR12* in the WES belongs to the GPCR1 family and it can promote neurite outgrowth and block myelin inhibition in neurons. *GRM7* and *GPR12* are all in the peptide ligand-binding receptor pathway that are related with GPCR signaling pathway. Both of them play an important role in cell metabolism and the expression of genes in brain. *SPTBN1* in the WES is an actin crosslinking and molecular scaffold protein that interacts with calmodulin in a calcium-dependent manner and is thus candidate for the calcium-dependent movement of the cytoskeleton at the membrane. (9) *SPTBN1* and *GRM7* are all involved in the signaling by the GPCR pathway. *MAP2K3* in the WES is a dual specificity protein kinase that participates in the MAP kinase-mediated signaling cascade. Importantly, *MAP2K3* and *GRM7* are both involved in three signal transduction pathways (Neuropathic pain-signaling/G-Beta Gamma signaling/CREB pathway) in the brain. The three pathways are all related to the GPCR signaling pathway. On the whole, findings from the GWAS and the WES can build up a pathway network where the *GRM7* is the central hub gene and the GPCR signaling pathway may be the main signal transduction pathway in pathophysiology of schizophrenia and response to risperidone.

Significantly, when we combined the findings of GWAS and WES with targets (*DRD2* and *HTR2A*) of risperidone, an extended pathway network was found. The *GRM7* and *MAP2K3* are involved in the G-Beta Gamma signaling with *DRD2* and *HTR2A*. The *GRM7* and *GPR12* participate in the peptide ligand-binding receptor pathway with these two targets. Furthermore, one of the target pathways of risperidone termed the neuroactive ligand-receptor interaction is part of the peptide ligand-binding receptor pathway.

## Discussion

To the best of our knowledge, this is the first study so far to identify both common variants with GWAS and rare variants with WES of treatment response to antipsychotics in patients with schizophrenia. We identified several SNPs associated with the treatment response to risperidone in the GWAS. The WES revealed four SNPs located in three genes showing genome-wide significant association with treatment response to risperidone and ten genes were significantly associated with response status in the gene-based association analyses.

Our results supported the hypothesis that the genetic architecture of drug response was likely to be similar to those of common diseases that were determined by a combination of multiple common and rare variants [29]. Several explanations for the genetic architecture of drug response are plausible. First, multiple variants contributing to drug response are segregating in the population at a wide allele frequency spectrum. Second, rare variants may impart larger effects on gene function, in addition to the variants with major impact, common variants with subtle effects also contribute to variation in drug response.

To identify the association between common SNPs and treatment response, we performed SNP-based association analysis through GWAS. First, we identified three novel *GRM7* SNPs (rs141134664, rs57521140, and rs73809055), with genome-wide significance. *GRM7* belongs to the G protein-coupled receptor family, a major excitatory neurotransmitter in the central nervous system that is involved in most aspects of normal brain function and can be perturbed in many neuropathologic conditions [30]. A SNP (rs2133450) in European populations and two SNPs (rs2069062 and rs2014195) in American populations located in this gene were reported to be associated with the effects of risperidone on the schizophrenia in previous GWASs of antipsychotic treatment [31, 32]. In particular, the rs2069062 has been included in the PharmGKB for clinical annotation. In addition, another SNP (rs9883258) was reported to be a potential marker for the therapeutic response of seven commonly used antipsychotic drugs in the Chinese Han population [33]. This trans-ethnic role of *GRM7* in treatment response in patients with schizophrenia boosts our confidence in the authenticity of the identified risk signals. *GRM7* is thus probably associated with risperidone treatment outcomes in patients with schizophrenia. We noticed that most of the 185 SNPs with genome-wide significance (87 of 185) were associated with *PRMT3* and the 87 SNPs were all in high linkage disequilibrium (LD) suggesting that these SNPs are more likely to be associated signals [34]. *PRMT3* belongs to the protein arginine methyltransferase family involved in catalyzing the transfer of methyl groups from S-adenosylmethionine to the arginine residues on histones and other proteins [35]. Our eQTL results suggested that the identified SNPs in this gene might affect treatment outcomes through drug metabolism, neurotransmitter, or neuronal development. We also found several other important genes that carried significantly associated SNPs, as described below. *USP48* may play a regulatory role at postsynaptic sites. This gene encodes a protein containing domains that associate it with peptidase family C19, also known as family 2 ubiquitin carboxyl-terminal hydrolases [36]. Furthermore, *USP48* and *PRMT3* are all involved in the Metabolism of Proteins pathway which may associated with drug metabolism. *SGCZ* is part of the sarcoglycan complex, a subcomplex of the dystrophin-glycoprotein complex that forms a link between the F-actin cytoskeleton and the extracellular matrix [37]. An SNP (rs1991346) in the *SGCZ* was associated with depressive symptom improvement and response to antidepressants [38]. Additionally, two SNPs (rs116939006 and rs146790986) in this gene were reported to be associated with schizophrenia and response to paliperidone [39, 40]. *DOCK5* encodes a member of the dedicator of cytokinesis protein family [41]. Members of this family act as guanine nucleotide exchange factors for small Rho family G proteins, which are important signal transduction molecules for many signaling pathways in the pathophysiological process of schizophrenia and the treatment response of antipsychotics [42]. *HTATIP2* is an oxidoreductase required for tumor suppression and may act as a redox sensor linked to transcription through regulation of nuclear import [43]. Our eQTL results indicated that the identified SNPs in this gene might affect the treatment response through a similar process in which *PRMT3* is involved. *PALM2AKAP2* belongs to the paralemmin down gene family which may have evolved contiguously with the paralemmin genes and are associated with other paralemmin paralogs in humans and several other taxa [44]. A SNP (rs4978848) in this gene was reported to be associated with cognition [45]. Therefore, although the SNP in this gene did not have eQTL effects in our study, further studies of its effects on *PALM2AKAP2*-related signals are justified.

For common SNPs tested in WES, we found four SNPs achieved genome-wide significance. Two SNPs (rs2271323 and rs2271326) were located in *SPTBN1*, which is a member of a family of beta-spectrin genes with functions in the determination of cell shape, arrangement of transmembrane proteins, and organization of organelles [46]. It was reported that *SPTBN1* interacted with calmodulin in a calcium-dependent manner, which was believed to have close link with the pathophysiological process of schizophrenia [47]. Another SNP (rs117462017) belongs to *PACC1* that can mediate import of chloride ion in response to extracellular acidic pH and is involved in acidosis-induced cell death by mediating chloride influx and subsequent cell swelling [48, 49]. The last SNP was a missense variant located in *HMGXB3*, which is one of the noncanonical high mobility group (HMG) genes. The encoded protein of this gene contains an HMG-box domain found in DNA binding proteins such as transcription factors and chromosomal proteins [50]. Although no study has reported the association between *HMGXB3* and the treatment response of antipsychotics or schizophrenia, this missense variant can affect the function of transcription factors which play an important role in transcription. Further studies of its effect on the treatment response of antipsychotics or schizophrenia are needed.

To identify the association between rare SNVs and the treatment response of antipsychotics, we performed gene-based association analysis using WES. We found *GPR12* with significance in our study. *GPR12* belongs to the GPCR family and can promote neurite outgrowth and block myelin inhibition in neurons [51]. It has been reported that *GPR12* was associated with response to antipsychotic drug [52]. We were aware of another GPCR family gene (*GRM7*) that was also significant in the GWAS results, which indicated that the signal transduction pathway mediated by GPCRs may play an important role in the pharmacodynamics and pharmacokinetics of antipsychotic drugs. Given remarkably similar results from the GWAS and WES, we are more convinced that the variance of treatment response among patients with schizophrenia emerged as the result of the interaction between common variants and rare variants. Another significant gene that we found was *MAP2K3*, which is a dual specificity protein kinase and belongs to the MAP kinase kinase family [53]. This kinase participates in the MAP kinase-mediated signaling cascade which is the downstream signal of the signal transduction pathway mediated by GPCR. Active MAPK is transferred into the nucleus and then phosphorylates some transcription factors [54]. The whole signal transduction pathway is believed to be associated with the pathophysiological process of schizophrenia and the treatment response of antipsychotics [42]. In addition, we also found eight other genes that passed the three tests in the gene-based association analysis. Although, we did not annotate any available evidence that can support the potential role of antipsychotics or schizophrenia in the treatment response, future studies may need to validate them.

We noticed a lack of shared identified loci between GWAS and WES, for which there are two possible explanations. First, although some pharmacogenomic effects tend to be larger and involve fewer genes than those detected in GWAS and WES studies for complex diseases, small sample sizes may still have insufficient power to detect ideal convergence. Second, the minor allele frequency distribution of variants for pharmacogenomics showed an obvious excess of low allele frequency variants and a significant number of drug response variants with high allele frequency in the population compared with common diseases and other complex traits. Multiple variants with a wide allele frequency spectrum contribute to drug response thus GWAS and WES may detect different signals.

Although a shared locus was not found between GWAS and WES, association signals tend to cluster into key pathways that drive treatment response etiology. We noticed that *GRM7* was associated with *SPTBN1*, *MAP2K3*, and *GPR12* in different signaling pathways. Our findings were supported by the core gene omnigenic model which assumes that most traits can be directly affected by a modest number of genes or gene pathways with specific roles in disease etiology, as well as their direct regulators [18]. These genes are “core genes”, such as *GRM7*, that tend to have biologically interpretable roles in treatment response. There are also a large number of genes without direct effects called “peripheral genes”, such as *SPTBN1*, *MAP2K3*, and *GPR12*, that are propagated through regulatory networks to a much smaller number of core genes [55]. The signaling pathways we identified were all associated with *DRD2* and *HTR2A* which are targets of risperidone. Drug pharmacokinetics and pharmacodynamics operate within networks of proteins that are responsible for drug metabolism, transport, and drug target. Genetic influences on drug response are frequently identified within these networks. Network analysis showed that GWAS- or WES-reported genes were close to drug target genes in a biological network [56, 57], and distributions showed that distances from a GWAS- or WES-reported gene to the closest drug targets were on average much shorter than those of a random gene to a closest drug target [58], which is consistent with our findings.

There are several limitations of the present study. First, although a large proportion of the current pharmacogenomic GWAS or WES findings have been reported with small sample sizes, such as ours, which might inflate the type I error rate and reduce power for detecting solidly associated genetic markers. Replication and extension of these findings are needed in studies with much larger samples. Second, nongenetic factors, such as environmental factors, duration of illness, and previous antipsychotics, which might affect interindividual differences in antipsychotic drug response, were not considered in our analyses. Third, although a significance threshold on the order of *P* < 1 × 10^-5^ is acceptable in pharmacogenomic studies, the risk of false positive results may exist when statistical power is low. Fourth, we provided a new research paradigm for identifying susceptibility loci for treatment response to antipsychotic drugs, but other omics technologies are also necessary to better characterize the joint contribution of variants and decipher the molecular pathways they affect.

In summary, we explored a GWAS and WES combined approach to comprehensively analyze the role of common and rare variants in the efficacy of risperidone in schizophrenic patients. We have identified several important loci and genes that are involved in the efficacy of risperidone. Our findings provide an illustration of the future potential of this approach in guiding the treatment of schizophrenia. The preliminary nature of our findings precludes our abilities to translate the findings into the prediction of clinical response. However, these findings warrant replications and should be extended with larger samples to confirm their use in the development of personalized medicine.

## Supplementary information

Supplementary materials

## References

[1] Thaker GK, Carpenter WT (2001). Advances in schizophrenia. Nat Med.

[2] McGrath J, Saha S, Chant D, Welham J (2008). Schizophrenia: a concise overview of incidence, prevalence, and mortality. Epidemiol Rev.

[3] Walker ER, McGee RE, Druss BG (2015). Mortality in mental disorders and global disease burden implications: a systematic review and meta-analysis. JAMA Psychiatry.

[4] Haddad PM, Correll CU (2018). The acute efficacy of antipsychotics in schizophrenia: a review of recent meta-analyses. Ther Adv Psychopharmacol.

[5] Lieberman JA, Stroup TS, McEvoy JP, Swartz MS, Rosenheck RA, Perkins DO (2005). Effectiveness of antipsychotic drugs in patients with chronic schizophrenia. N Engl J Med.

[6] Cloutier M, Aigbogun MS, Guerin A, Nitulescu R, Ramanakumar AV, Kamat SA (2016). The Economic Burden of Schizophrenia in the United States in 2013. J Clin Psychiatry.

[7] Pennington M, McCrone P (2017). The Cost of Relapse in Schizophrenia. Pharmacoeconomics..

[8] Bousman CA, Bengesser SA, Aitchison KJ, Amare AT, Aschauer H, Baune BT (2021). Review and consensus on pharmacogenomic testing in psychiatry. Pharmacopsychiatry.

[9] McCutcheon RA, Krystal JH, Howes OD (2020). Dopamine and glutamate in schizophrenia: biology, symptoms and treatment. World Psychiatry.

[10] Yu H, Yan H, Wang L, Li J, Tan L, Deng W (2018). Five novel loci associated with antipsychotic treatment response in patients with schizophrenia: a genome-wide association study. Lancet Psychiatry.

[11] van Calker D, Serchov T (2021). The “missing heritability”-Problem in psychiatry: Is the interaction of genetics, epigenetics and transposable elements a potential solution?. Neurosci Biobehav Rev.

[12] Manolio TA, Collins FS, Cox NJ, Goldstein DB, Hindorff LA, Hunter DJ (2009). Finding the missing heritability of complex diseases. Nature.

[13] Park JH, Wacholder S, Gail MH, Peters U, Jacobs KB, Chanock SJ (2010). Estimation of effect size distribution from genome-wide association studies and implications for future discoveries. Nat Genet.

[14] Hirschhorn JN (2009). Genomewide association studies–illuminating biologic pathways. N Engl J Med.

[15] Fukunaga K, Momozawa Y, Mushiroda T (2021). Update on next generation sequencing of pharmacokinetics-related genes: development of the PKseq panel, a platform for amplicon sequencing of drug-metabolizing enzyme and drug transporter genes. Drug Metab Pharmacokinet.

[16] Wang Q, Man Wu H, Yue W, Yan H, Zhang Y, Tan L (2018). Effect of damaging rare mutations in synapse-related gene sets on response to short-term antipsychotic medication in chinese patients with schizophrenia: a randomized clinical trial. JAMA Psychiatry.

[17] Islam F, Men X, Yoshida K, Zai CC, Müller DJ (2021). Pharmacogenetics-guided advances in antipsychotic treatment. Clin Pharm Ther.

[18] Boyle EA, Li YI, Pritchard JK (2017). An expanded view of complex traits: from polygenic to omnigenic. Cell.

[19] Fabbri C, Kasper S, Kautzky A, Zohar J, Souery D, Montgomery S (2020). A polygenic predictor of treatment-resistant depression using whole exome sequencing and genome-wide genotyping. Transl Psychiatry.

[20] Chaplin M, Kirkham JJ, Dwan K, Sloan DJ, Davies G, Jorgensen AL (2020). Strengthening the reporting of pharmacogenetic studies: development of the STROPS guideline. PLoS Med.

[21] Delaneau O, Zagury JF, Marchini J (2013). Improved whole-chromosome phasing for disease and population genetic studies. Nat Methods.

[22] Howie BN, Donnelly P, Marchini J (2009). A flexible and accurate genotype imputation method for the next generation of genome-wide association studies. PLoS Genet.

[23] Daly AK (2010). Genome-wide association studies in pharmacogenomics. Nat Rev Genet.

[24] Mu G, Xiang Q, Zhang Z, Liu C, Zhang H, Liu Z (2020). PNPT1 and PCGF3 variants associated with angiotensin-converting enzyme inhibitor-induced cough: a nested case-control genome-wide study. Pharmacogenomics.

[25] The GTEx Consortium atlas of genetic regulatory effects across human tissues. Science. 2020;369:1318–30. 10.1126/science.aaz1776 PMC7737656 32913098

[26] Ramasamy A, Trabzuni D, Guelfi S, Varghese V, Smith C, Walker R (2014). Genetic variability in the regulation of gene expression in ten regions of the human brain. Nat Neurosci.

[27] Asimit J, Zeggini E (2010). Rare variant association analysis methods for complex traits. Annu Rev Genet.

[28] Zhan X, Hu Y, Li B, Abecasis GR, Liu DJ (2016). RVTESTS: an efficient and comprehensive tool for rare variant association analysis using sequence data. Bioinformatics.

[29] Roden DM, Wilke RA, Kroemer HK, Stein CM (2011). Pharmacogenomics: the genetics of variable drug responses. Circulation.

[30] Song JM, Kang M, Park DH, Park S, Lee S, Suh YH (2021). Pathogenic GRM7 mutations associated with neurodevelopmental disorders impair axon outgrowth and presynaptic terminal development. J Neurosci.

[31] Sacchetti E, Magri C, Minelli A, Valsecchi P, Traversa M, Calza S (2017). The GRM7 gene, early response to risperidone, and schizophrenia: a genome-wide association study and a confirmatory pharmacogenetic analysis. Pharmacogenomics J.

[32] Stevenson JM, Reilly JL, Harris MS, Patel SR, Weiden PJ, Prasad KM (2016). Antipsychotic pharmacogenomics in first episode psychosis: a role for glutamate genes. Transl Psychiatry.

[33] Liang W, Yu H, Su Y, Lu T, Yan H, Yue W (2020). Variants of GRM7 as risk factor and response to antipsychotic therapy in schizophrenia. Transl Psychiatry.

[34] Bulik-Sullivan BK, Loh PR, Finucane HK, Ripke S, Yang J, Patterson N (2015). LD Score regression distinguishes confounding from polygenicity in genome-wide association studies. Nat Genet.

[35] Guccione E, Richard S (2019). The regulation, functions and clinical relevance of arginine methylation. Nat Rev Mol Cell Biol.

[36] Bassani S, van Beelen E, Rossel M, Voisin N, Morgan A, Arribat Y (2021). Variants in USP48 encoding ubiquitin hydrolase are associated with autosomal dominant non-syndromic hereditary hearing loss. Hum Mol Genet.

[37] Wheeler MT, Zarnegar S, McNally EM (2002). Zeta-sarcoglycan, a novel component of the sarcoglycan complex, is reduced in muscular dystrophy. Hum Mol Genet.

[38] Fabbri C, Kasper S, Kautzky A, Bartova L, Dold M, Zohar J (2019). Genome-wide association study of treatment-resistance in depression and meta-analysis of three independent samples. Br J Psychiatry.

[39] Lam M, Hill WD, Trampush JW, Yu J, Knowles E, Davies G (2019). Pleiotropic meta-analysis of cognition, education, and schizophrenia differentiates roles of early neurodevelopmental and adult synaptic pathways. Am J Hum Genet.

[40] Li Q, Wineinger NE, Fu DJ, Libiger O, Alphs L, Savitz A (2017). Genome-wide association study of paliperidone efficacy. Pharmacogenet Genomics.

[41] Sanders MA, Ampasala D, Basson MD (2009). DOCK5 and DOCK1 regulate Caco-2 intestinal epithelial cell spreading and migration on collagen IV. J Biol Chem.

[42] Boczek T, Mackiewicz J, Sobolczyk M, Wawrzyniak J, Lisek M, Ferenc B, et al. The role of G protein-coupled receptors (GPCRs) and calcium signaling in Schizophrenia. Focus on GPCRs activated by neurotransmitters and chemokines. Cells. 2021;10:1228. 10.3390/cells10051228 PMC8155952 34067760

[43] Li M, Li J, Guo X, Pan H, Zhou Q Absence of HTATIP2 expression in A549 lung adenocarcinoma cells promotes tumor plasticity in response to hypoxic stress. Cancers. 2020;12:1538. 10.3390/cancers12061538 PMC7352940 32545251

[44] Hultqvist G, Ocampo Daza D, Larhammar D, Kilimann MW (2012). Evolution of the vertebrate paralemmin gene family: ancient origin of gene duplicates suggests distinct functions. PLoS One.

[45] Cox AJ, Hugenschmidt CE, Raffield LM, Langefeld CD, Freedman BI, Williamson JD (2014). Heritability and genetic association analysis of cognition in the Diabetes Heart Study. Neurobiol Aging.

[46] Zhi X, Lin L, Yang S, Bhuvaneshwar K, Wang H, Gusev Y (2015). βII-Spectrin (SPTBN1) suppresses progression of hepatocellular carcinoma and Wnt signaling by regulation of Wnt inhibitor kallistatin. Hepatology.

[47] Lintunen J, Lähteenvuo M, Tiihonen J, Tanskanen A, Taipale H (2021). Adenosine modulators and calcium channel blockers as add-on treatment for schizophrenia. NPJ Schizophr.

[48] Yang J, Chen J, Del Carmen Vitery M, Osei-Owusu J, Chu J, Yu H (2019). PAC, an evolutionarily conserved membrane protein, is a proton-activated chloride channel. Science.

[49] Ullrich F, Blin S, Lazarow K, Daubitz T, von Kries JP, Jentsch TJ Identification of TMEM206 proteins as pore of PAORAC/ASOR acid-sensitive chloride channels. Elife. 2019;8:e49187. 10.7554/eLife.49187 PMC6663466 31318332

[50] Sönmezer C, Kleinendorst R, Imanci D, Barzaghi G, Villacorta L, Schübeler D (2021). Molecular co-occupancy identifies transcription factor binding cooperativity in vivo. Mol Cell.

[51] Allende G, Chávez-Reyes J, Guerrero-Alba R, Vázquez-León P, Marichal-Cancino BA (2020). Advances in neurobiology and pharmacology of GPR12. Front Pharm.

[52] Drago A, Kure Fischer E (2018). A molecular pathway analysis informs the genetic risk for arrhythmias during antipsychotic treatment. Int Clin Psychopharmacol.

[53] Brancho D, Tanaka N, Jaeschke A, Ventura JJ, Kelkar N, Tanaka Y (2003). Mechanism of p38 MAP kinase activation in vivo. Genes Dev.

[54] Grimsey NJ, Lin Y, Narala R, Rada CC, Mejia-Pena H, Trejo J (2019). G protein-coupled receptors activate p38 MAPK via a non-canonical TAB1-TAB2- and TAB1-TAB3-dependent pathway in endothelial cells. J Biol Chem.

[55] Wray NR, Wijmenga C, Sullivan PF, Yang J, Visscher PM (2018). Common disease is more complex than implied by the core gene omnigenic model. Cell.

[56] Yeh SH, Yeh HY, Soo VW (2012). A network flow approach to predict drug targets from microarray data, disease genes and interactome network - case study on prostate cancer. J Clin Bioinforma.

[57] Jia P, Wang L, Fanous AH, Pato CN, Edwards TL, Zhao Z (2012). Network-assisted investigation of combined causal signals from genome-wide association studies in schizophrenia. PLoS Comput Biol.

[58] Rossin EJ, Lage K, Raychaudhuri S, Xavier RJ, Tatar D, Benita Y (2011). Proteins encoded in genomic regions associated with immune-mediated disease physically interact and suggest underlying biology. PLoS Genet.

