# Supplementary material for: Different responses to risperidone treatment in Schizophrenia: a multicenter genome-wide association and whole exome sequencing joint study
Source: Transl Psychiatry. 2022 Apr 28;12:173. doi: 10.1038/s41398-022-01942-w (PMC9050705; doi:10.1038/s41398-022-01942-w)

Supplementary table S1. Genomic regions of non-intergenic variants with P < 5×10^-5^ in the Genome-wide association of risperidone treatment

| CHR | SNP | Position | A1 | A2 | Gene | variant location | BETA | P |
| --- | --- | --- | --- | --- | --- | --- | --- | --- |
| 2 | rs6433797 | 180440141 | C | T | *ZNF385B* | intron variant | 25.95 | 1.24E-05 |
| 20 | rs8126177 | 44102896 | T | C | *WFDC2* | intron variant | 6.518 | 1.58E-05 |
| 6 | rs1408737 | 23764927 | C | C | *WFDC2* | intron variant | 8.629 | 2.22E-05 |
| 20 | rs75766050 | 44098542 | A | G | *WFDC2* | intron variant | 6.294 | 2.87E-05 |
| 20 | rs74925607 | 44103792 | T | G | *WFDC2* | intron variant | 6.294 | 2.87E-05 |
| 20 | rs6104173 | 44096559 | T | C | *WFDC2* | upstream gene variant | 6.294 | 2.87E-05 |
| 20 | rs6017578 | 44101129 | A | G | *WFDC2* | intron variant | 6.294 | 2.87E-05 |
| 13 | rs80331970 | 99105022 | C | T | *STK24* | 3 prime UTR variant | -25.18 | 2.05E-05 |
| 13 | rs80285975 | 99099284 | A | G | *STK24* | 3 prime UTR variant | -25.18 | 2.05E-05 |
| 13 | rs79063125 | 99102946 | G | C | *STK24* | 3 prime UTR variant | -25.18 | 2.05E-05 |
| 13 | rs78714822 | 99101389 | A | C | *STK24* | 3 prime UTR variant | -25.18 | 2.05E-05 |
| 13 | rs77027456 | 99106990 | C | T | *STK24* | intron variant | -25.18 | 2.05E-05 |
| 13 | rs75029013 | 99109653 | A | G | *STK24* | intron variant | -25.18 | 2.05E-05 |
| 13 | rs3783004 | 99112125 | T | G | *STK24* | intron variant | -25.18 | 2.05E-05 |
| 18 | rs147081160 | 36973109 | C | T | *STK24* | intron variant | 15.46 | 2.49E-05 |
| 17 | rs139116684 | 39137090 | T | G | *SKAP1* | intron variant | 22.37 | 2.94E-05 |
| 1 | rs115688407 | 223211495 | A | G | *SGCZ* | intron variant | -10.46 | 1.43E-05 |
| 8 | rs74435630 | 14496536 | A | G | *SGCZ* | intron variant | 9.995 | 2.73E-05 |
| 8 | rs80030371 | 14487037 | T | G | *SGCZ* | intron variant | 9.926 | 2.99E-05 |
| 8 | rs79858633 | 14496492 | A | C | *SGCZ* | intron variant | 9.926 | 2.99E-05 |
| 8 | rs79418606 | 14495204 | G | A | *SGCZ* | intron variant | 9.926 | 2.99E-05 |
| 8 | rs78897063 | 14495936 | C | A | *SGCZ* | intron variant | 9.926 | 2.99E-05 |
| 8 | rs78735285 | 14497504 | T | C | *SGCZ* | intron variant | 9.926 | 2.99E-05 |
| 8 | rs78041224 | 14493721 | G | T | *SGCZ* | intron variant | 9.926 | 2.99E-05 |
| 8 | rs77998946 | 14480212 | C | A | *SGCZ* | intron variant | 9.926 | 2.99E-05 |
| 8 | rs77601595 | 14483156 | C | T | *SGCZ* | intron variant | 9.926 | 2.99E-05 |
| 8 | rs77571706 | 14486313 | G | C | *SGCZ* | intron variant | 9.926 | 2.99E-05 |
| 8 | rs76923285 | 14496404 | C | T | *SGCZ* | intron variant | 9.926 | 2.99E-05 |
| 8 | rs76678829 | 14497333 | A | T | *SGCZ* | intron variant | 9.926 | 2.99E-05 |
| 8 | rs76567643 | 14495401 | A | G | *SGCZ* | intron variant | 9.926 | 2.99E-05 |
| 8 | rs76460209 | 14496664 | G | C | *SGCZ* | intron variant | 9.926 | 2.99E-05 |
| 8 | rs76376302 | 14495031 | T | C | *SGCZ* | intron variant | 9.926 | 2.99E-05 |
| 8 | rs75961994 | 14493869 | T | C | *SGCZ* | intron variant | 9.926 | 2.99E-05 |
| 8 | rs75660738 | 14495951 | G | C | *SGCZ* | intron variant | 9.926 | 2.99E-05 |
| 8 | rs74967391 | 14493703 | G | C | *SGCZ* | intron variant | 9.926 | 2.99E-05 |
| 8 | rs74579775 | 14494983 | T | T | *SGCZ* | intron variant | 9.926 | 2.99E-05 |
| 8 | rs74578509 | 14497729 | A | A | *SGCZ* | intron variant | 9.926 | 2.99E-05 |
| 8 | rs74390572 | 14495897 | T | A | *SGCZ* | intron variant | 9.926 | 2.99E-05 |
| 8 | rs6983709 | 14492121 | G | A | *SGCZ* | intron variant | 9.926 | 2.99E-05 |
| 8 | rs192979028 | 14487518 | T | C | *SGCZ* | intron variant | 9.926 | 2.99E-05 |
| 8 | rs186995437 | 14487517 | A | A | *SGCZ* | intron variant | 9.926 | 2.99E-05 |
| 8 | rs1510438 | 14494860 | C | C | *SGCZ* | intron variant | 9.926 | 2.99E-05 |
| 8 | rs1510433 | 14483355 | T | A | *SGCZ* | intron variant | 9.926 | 2.99E-05 |
| 9 | rs146158414 | 112823027 | G | A | *SERINC2* | upstream gene variant | 26.11 | 1.36E-05 |
| 9 | rs140493905 | 112823227 | G | C | *SDK2* | intron variant | 26.11 | 1.36E-05 |
| 7 | rs2538916 | 88299566 | T | C | *RNU6-847P* | upstream gene variant | -6.997 | 1.63E-05 |
| 18 | rs7729212 | 4795000 | A | G | *RACK1* | upstream gene variant | -20.05 | 1.15E-05 |
| 11 | rs4630292 | 20531949 | A | G | *PRMT3* | downstream gene variant | 4.723 | 1.07E-05 |
| 11 | rs7395007 | 20522290 | G | A | *PRMT3* | intron variant | -4.945 | 1.09E-05 |
| 11 | rs7104346 | 20516027 | A | G | *PRMT3* | intron variant | -4.945 | 1.09E-05 |
| 18 | rs14651706 | 4780232 | T | G | *PRMT3* | intron variant | -20.05 | 1.15E-05 |
| 10 | rs2504241 | 35205642 | T | C | *PRMT3* | intron variant | 6.226 | 1.17E-05 |
| 11 | rs736283 | 20510864 | C | T | *PRMT3* | intron variant | -4.876 | 1.35E-05 |
| 11 | rs2403600 | 20520461 | T | G | *PRMT3* | intron variant | -4.876 | 1.35E-05 |
| 6 | rs10806973 | 23751135 | G | A | *PRMT3* | upstream gene variant | 8.724 | 1.39E-05 |
| 11 | rs7927653 | 20516344 | A | T | *PRMT3* | intron variant | -4.867 | 1.42E-05 |
| 1 | rs114709668 | 223211494 | C | T | *PRMT3* | intron variant | -10.46 | 1.43E-05 |
| 11 | rs737183 | 20508195 | T | T | *PRMT3* | intron variant | -4.861 | 1.77E-05 |
| 11 | rs7479181 | 20424775 | G | T | *PRMT3* | intron variant | 4.895 | 1.83E-05 |
| 11 | rs7127831 | 20414096 | G | C | *PRMT3* | intron variant | 4.887 | 1.87E-05 |
| 11 | rs6483673 | 20423809 | A | G | *PRMT3* | intron variant | 4.887 | 1.87E-05 |
| 11 | rs7479049 | 20433483 | G | A | *PRMT3* | intron variant | 4.835 | 1.97E-05 |
| 11 | rs6416026 | 20432808 | G | C | *PRMT3* | intron variant | 4.835 | 1.97E-05 |
| 11 | rs7114918 | 20478309 | T | G | *PRMT3* | intron variant | 4.846 | 1.98E-05 |
| 11 | rs10741838 | 20447419 | C | T | *PRMT3* | intron variant | 4.828 | 2.01E-05 |
| 6 | rs10946626 | 23755828 | A | A | *PRMT3* | intron variant | 8.629 | 2.22E-05 |
| 6 | rs10806975 | 23766367 | A | A | *PRMT3* | intron variant | 8.629 | 2.22E-05 |
| 6 | rs10806974 | 23755748 | A | A | *PRMT3* | intron variant | 8.629 | 2.22E-05 |
| 6 | rs10755630 | 23755654 | C | C | *PRMT3* | intron variant | 8.629 | 2.22E-05 |
| 6 | rs10755629 | 23755641 | G | G | *PRMT3* | intron variant | 8.629 | 2.22E-05 |
| 6 | rs10755628 | 23755635 | T | T | *PRMT3* | intron variant | 8.629 | 2.22E-05 |
| 6 | rs10456286 | 23767741 | C | C | *PRMT3* | intron variant | 8.629 | 2.22E-05 |
| 6 | rs10946632 | 23776976 | G | G | *PRMT3* | downstream gene variant | 8.563 | 2.60E-05 |
| 20 | rs6094144 | 44087619 | A | T | *PIGT* | downstream gene variant | 6.584 | 1.83E-05 |
| 20 | rs6094143 | 44087618 | T | A | *PIGT* | downstream gene variant | 6.584 | 1.83E-05 |
| 20 | rs66797225 | 44087833 | C | T | *PIGT* | downstream gene variant | 6.502 | 2.03E-05 |
| 18 | rs14933295 | 4778394 | G | A | *PALM2AKAP2* | intron variant | -20.05 | 1.15E-05 |
| 18 | rs14899173 | 4779876 | T | C | *PALM2AKAP2* | intron variant | -20.05 | 1.15E-05 |
| 9 | rs80344044 | 112823678 | T | G | *PALM2AKAP2* | intron variant | 26.11 | 1.36E-05 |
| 9 | rs79710378 | 112804267 | T | C | *PALM2AKAP2* | intron variant | 26.11 | 1.36E-05 |
| 9 | rs78969175 | 112814900 | A | C | *PALM2AKAP2* | intron variant | 26.11 | 1.36E-05 |
| 9 | rs7872174 | 112813702 | C | T | *PALM2AKAP2* | intron variant | 26.11 | 1.36E-05 |
| 9 | rs78083977 | 112823638 | G | A | *PALM2AKAP2* | intron variant | 26.11 | 1.36E-05 |
| 9 | rs77487126 | 112822111 | G | A | *PALM2AKAP2* | intron variant | 26.11 | 1.36E-05 |
| 9 | rs76519535 | 112820707 | G | C | *PALM2AKAP2* | intron variant | 26.11 | 1.36E-05 |
| 9 | rs76235006 | 112804428 | T | C | *PALM2AKAP2* | intron variant | 26.11 | 1.36E-05 |
| 9 | rs74828737 | 112822194 | T | C | *PALM2AKAP2* | intron variant | 26.11 | 1.36E-05 |
| 9 | rs7043272 | 112811603 | T | G | *PALM2AKAP2* | intron variant | 26.11 | 1.36E-05 |
| 9 | rs56375930 | 112827270 | T | G | *PALM2AKAP2* | intron variant | 26.11 | 1.36E-05 |
| 9 | rs191372192 | 112822681 | C | G | *PALM2AKAP2* | intron variant | 26.11 | 1.36E-05 |
| 9 | rs190097702 | 112821104 | A | G | *PALM2AKAP2* | intron variant | 26.11 | 1.36E-05 |
| 9 | rs185776417 | 112821103 | G | A | *PALM2AKAP2* | intron variant | 26.11 | 1.36E-05 |
| 9 | rs151067204 | 112827716 | G | A | *PALM2AKAP2* | intron variant | 26.11 | 1.36E-05 |
| 9 | rs148568128 | 112823006 | T | C | *PALM2AKAP2* | intron variant | 26.11 | 1.36E-05 |
| 9 | rs146217815 | 112821282 | T | C | *PALM2AKAP2* | intron variant | 26.11 | 1.36E-05 |
| 6 | rs1408741 | 23746073 | T | T | *PALM2AKAP2* | intron variant | 8.724 | 1.39E-05 |
| 1 | rs116960345 | 223208453 | T | C | *PALM2AKAP2* | intron variant | -10.63 | 1.66E-05 |
| 13 | rs144790743 | 99108614 | C | T | *PALM2AKAP2* | intron variant | -25.18 | 2.05E-05 |
| 6 | rs1408738 | 23764940 | A | A | *PALM2AKAP2* | intron variant | 8.629 | 2.22E-05 |
| 6 | rs1321896 | 23759400 | C | C | *PALM2AKAP2* | intron variant | 8.629 | 2.22E-05 |
| 3 | rs1505573 | 26908181 | C | G | *PALM2AKAP2* | intron variant | 5.409 | 2.43E-05 |
| 9 | rs149038184 | 112829683 | A | G | *MIR924HG* | intron variant, noncoding transcript variant | 26.11 | 1.36E-05 |
| 6 | rs10456285 | 23765890 | T | T | *MINAR1* | synonymous variant | 8.629 | 2.22E-05 |
| 1 | rs79527954 | 222102517 | T | A | *LINC02257* | intron variant, noncoding transcript variant | -12.45 | 1.91E-05 |
| 1 | rs79070709 | 222104813 | T | A | *LINC02257* | intron variant, noncoding transcript variant | -12.45 | 1.91E-05 |
| 1 | rs76321108 | 222111572 | T | C | *LINC02257* | intron variant, noncoding transcript variant | -12.45 | 1.91E-05 |
| 1 | rs75219127 | 222109480 | C | T | *LINC02257* | intron variant, noncoding transcript variant | -12.45 | 1.91E-05 |
| 1 | rs75104854 | 222104721 | T | A | *LINC02257* | intron variant, noncoding transcript variant | -12.45 | 1.91E-05 |
| 13 | rs117725897 | 44475004 | T | C | *LINC02257* | intron variant, noncoding transcript variant | 10.04 | 1.98E-05 |
| 9 | rs139910608 | 112816608 | C | G | *KRT40* | missense variant | 26.11 | 1.36E-05 |
| 9 | rs140022103 | 111216166 | C | T | *KRT40* | missense variant | 24.94 | 3.01E-05 |
| 1 | rs4344325 | 14993431 | C | T | *KAZN* | intron variant | 6.193 | 2.45E-05 |
| 1 | rs2038164 | 14973801 | T | C | *KAZN* | intron variant | 6.47 | 2.49E-05 |
| 11 | rs10766680 | 20516840 | A | A | *HTATIP2* | intron variant | -4.945 | 1.09E-05 |
| 11 | rs10766667 | 20412941 | A | G | *HTATIP2* | intron variant | 4.861 | 2.34E-05 |
| 1 | rs34780349 | 179726805 | T | G | *FAM163A* | intron variant | 24.94 | 2.68E-05 |
| 4 | rs62308565 | 56822757 | C | T | *CEP135* | intron variant | 10.68 | 1.11E-05 |
| 4 | rs3806749 | 56830934 | A | G | *CEP135* | intron variant | 10.61 | 1.27E-05 |
| 13 | rs74619788 | 44439347 | T | C | *CCDC122* | intron variant | 9.581 | 2.96E-05 |
| 16 | rs222140 | 15568243 | G | A | *BMERB1* | intron variant | 17.39 | 1.52E-05 |
| 6 | rs2328788 | 23736590 | T | T | *BMERB1* | intron variant | 8.636 | 1.78E-05 |
| 17 | rs76845320 | 31508251 | G | C | *ASIC2* | intron variant | 20.7 | 3.06E-05 |
| 17 | rs74719363 | 31523479 | T | G | *ASIC2* | intron variant | 20.7 | 3.06E-05 |
| 9 | rs115239371 | 112826329 | T | C | *ADAMTSL1* | intron variant | 26.11 | 1.36E-05 |
| 9 | rs79502831 | 18537863 | T | C | *ADAMTSL1* | intron variant | -15.2 | 1.74E-05 |
| 9 | rs78341308 | 18543859 | G | T | *ADAMTSL1* | intron variant | -15.2 | 1.74E-05 |
| 9 | rs78025895 | 18543983 | G | T | *ADAMTSL1* | intron variant | -15.2 | 1.74E-05 |
| 9 | rs77806323 | 18539025 | T | C | *ADAMTSL1* | intron variant | -15.2 | 1.74E-05 |
| 9 | rs77703658 | 18537701 | G | T | *ADAMTSL1* | intron variant | -15.2 | 1.74E-05 |
| 9 | rs76101324 | 18550350 | A | G | *ADAMTSL1* | intron variant | -15.2 | 1.74E-05 |
| 9 | rs73644234 | 18538031 | T | C | *ADAMTSL1* | intron variant | -15.2 | 1.74E-05 |
| 9 | rs61364591 | 18536514 | T | A | *ADAMTSL1* | intron variant | -15.2 | 1.74E-05 |
| 9 | rs61210955 | 18540456 | T | A | *ADAMTSL1* | intron variant | -15.2 | 1.74E-05 |
| 9 | rs59401514 | 18536649 | T | A | *ADAMTSL1* | intron variant | -15.2 | 1.74E-05 |
| 11 | rs2403591 | 20411503 | A | G | *ADAMTSL1* | intron variant | 4.887 | 1.87E-05 |
| 20 | rs139909077 | 44105448 | G | C | *ADAMTSL1* | intron variant | 6.294 | 2.87E-05 |

CHR chromosome, SNP single-nucleotide polymorphism, A1 Minor allele, A2 Major allele.

Supplementary table S2. Genomic regions with P < 1×10^-5^ in the Genome-wide association of risperidone treatment

| CHR | SNPs | Position | A1 | A2 | Gene | variant location | BETA | P |
| --- | --- | --- | --- | --- | --- | --- | --- | --- |
| 1 | rs16825842 | 21702311 | C | T | *USP48* | intron variant | 11.94 | 5.70E-06 |
| 1 | rs116165545 | 21754863 | A | G | *USP48* | intron variant | 11.47 | 9.33E-06 |
| 8 | rs78076666 | 14647881 | T | C | *SGCZ* | intron variant | 11.37 | 6.80E-06 |
| 11 | rs7950585 | 20401629 | C | T | *PRMT3* | intron variant | 5.325 | 2.37E-06 |
| 11 | rs7932359 | 20401146 | A | G | *PRMT3* | intron variant | 5.325 | 2.37E-06 |
| 11 | rs6483672 | 20401558 | A | G | *PRMT3* | intron variant | 5.325 | 2.37E-06 |
| 11 | rs6483671 | 20401189 | G | A | *PRMT3* | intron variant | 5.325 | 2.37E-06 |
| 11 | rs10833318 | 20400955 | A | G | *PRMT3* | intron variant | 5.325 | 2.37E-06 |
| 11 | rs10833319 | 20402780 | G | A | *PRMT3* | intron variant | 5.271 | 3.16E-06 |
| 11 | rs11025540 | 20386590 | C | G | *PRMT3* | upstream gene variant | 5.228 | 3.22E-06 |
| 11 | rs7948149 | 20397883 | C | A | *PRMT3* | intron variant | 5.264 | 3.23E-06 |
| 11 | rs7937501 | 20389872 | C | G | *PRMT3* | intron variant | 5.264 | 3.23E-06 |
| 11 | rs7937204 | 20389878 | G | A | *PRMT3* | intron variant | 5.264 | 3.23E-06 |
| 11 | rs7932200 | 20392017 | C | T | *PRMT3* | intron variant | 5.264 | 3.23E-06 |
| 11 | rs7932183 | 20391976 | C | T | *PRMT3* | intron variant | 5.264 | 3.23E-06 |
| 11 | rs7395927 | 20394089 | C | T | *PRMT3* | intron variant | 5.264 | 3.23E-06 |
| 11 | rs7394821 | 20394435 | C | A | *PRMT3* | intron variant | 5.264 | 3.23E-06 |
| 11 | rs6483670 | 20397410 | C | T | *PRMT3* | intron variant | 5.264 | 3.23E-06 |
| 11 | rs6483669 | 20396706 | G | A | *PRMT3* | intron variant | 5.264 | 3.23E-06 |
| 11 | rs2132966 | 20389094 | G | A | *PRMT3* | intron variant | 5.264 | 3.23E-06 |
| 11 | rs10766669 | 20399791 | T | C | *PRMT3* | intron variant | 5.264 | 3.23E-06 |
| 11 | rs10766668 | 20398159 | A | G | *PRMT3* | intron variant | 5.264 | 3.23E-06 |
| 11 | rs7943125 | 20460298 | C | T | *PRMT3* | intron variant | 5.208 | 3.44E-06 |
| 11 | rs7482946 | 20416732 | G | A | *PRMT3* | intron variant | 5.208 | 3.44E-06 |
| 11 | rs7479752 | 20409346 | C | T | *PRMT3* | intron variant | 5.208 | 3.44E-06 |
| 11 | rs7479021 | 20411804 | G | A | *PRMT3* | intron variant | 5.208 | 3.44E-06 |
| 11 | rs7478707 | 20414372 | C | T | *PRMT3* | intron variant | 5.208 | 3.44E-06 |
| 11 | rs7125358 | 20460877 | G | T | *PRMT3* | intron variant | 5.208 | 3.44E-06 |
| 11 | rs7117347 | 20462616 | G | A | *PRMT3* | intron variant | 5.208 | 3.44E-06 |
| 11 | rs7116248 | 20407407 | T | C | *PRMT3* | intron variant | 5.208 | 3.44E-06 |
| 11 | rs7102355 | 20415068 | G | T | *PRMT3* | intron variant | 5.208 | 3.44E-06 |
| 11 | rs6483690 | 20469540 | G | C | *PRMT3* | intron variant | 5.208 | 3.44E-06 |
| 11 | rs6483685 | 20437460 | A | T | *PRMT3* | intron variant | 5.208 | 3.44E-06 |
| 11 | rs6483683 | 20436952 | T | G | *PRMT3* | intron variant | 5.208 | 3.44E-06 |
| 11 | rs6416028 | 20411346 | A | G | *PRMT3* | intron variant | 5.208 | 3.44E-06 |
| 11 | rs6416027 | 20411327 | C | A | *PRMT3* | intron variant | 5.208 | 3.44E-06 |
| 11 | rs61876931 | 20413079 | C | T | *PRMT3* | intron variant | 5.208 | 3.44E-06 |
| 11 | rs3758804 | 20462430 | C | T | *PRMT3* | intron variant | 5.208 | 3.44E-06 |
| 11 | rs1160443 | 20447246 | G | A | *PRMT3* | intron variant | 5.208 | 3.44E-06 |
| 11 | rs11512079 | 20463624 | G | C | *PRMT3* | intron variant | 5.208 | 3.44E-06 |
| 11 | rs11025553 | 20408929 | C | T | *PRMT3* | intron variant | 5.208 | 3.44E-06 |
| 11 | rs10833331 | 20469158 | G | A | *PRMT3* | intron variant | 5.208 | 3.44E-06 |
| 11 | rs10766677 | 20471443 | C | G | *PRMT3* | intron variant | 5.208 | 3.44E-06 |
| 11 | rs10766671 | 20413194 | A | T | *PRMT3* | intron variant | 5.208 | 3.44E-06 |
| 11 | rs10766670 | 20407633 | G | A | *PRMT3* | intron variant | 5.208 | 3.44E-06 |
| 11 | rs10430902 | 20466023 | A | C | *PRMT3* | intron variant | 5.208 | 3.44E-06 |
| 11 | rs1036925 | 20466338 | C | T | *PRMT3* | intron variant | 5.208 | 3.44E-06 |
| 11 | rs1036924 | 20466690 | C | A | *PRMT3* | intron variant | 5.208 | 3.44E-06 |
| 11 | rs1001669 | 20452109 | G | T | *PRMT3* | intron variant | 5.208 | 3.44E-06 |
| 11 | rs957500 | 20428145 | A | C | *PRMT3* | intron variant | 5.202 | 3.50E-06 |
| 11 | rs7946612 | 20477268 | G | A | *PRMT3* | intron variant | 5.202 | 3.50E-06 |
| 11 | rs7938254 | 20437719 | G | A | *PRMT3* | intron variant | 5.202 | 3.50E-06 |
| 11 | rs7929063 | 20415811 | C | T | *PRMT3* | intron variant | 5.202 | 3.50E-06 |
| 11 | rs7482999 | 20416930 | G | A | *PRMT3* | intron variant | 5.202 | 3.50E-06 |
| 11 | rs7481390 | 20414565 | T | C | *PRMT3* | intron variant | 5.202 | 3.50E-06 |
| 11 | rs7127579 | 20422560 | G | A | *PRMT3* | intron variant | 5.202 | 3.50E-06 |
| 11 | rs7122192 | 20433955 | G | A | *PRMT3* | intron variant | 5.202 | 3.50E-06 |
| 11 | rs7121685 | 20430333 | C | T | *PRMT3* | intron variant | 5.202 | 3.50E-06 |
| 11 | rs7105438 | 20430260 | G | A | *PRMT3* | intron variant | 5.202 | 3.50E-06 |
| 11 | rs6483694 | 20476390 | A | T | *PRMT3* | intron variant | 5.202 | 3.50E-06 |
| 11 | rs6483681 | 20436195 | T | C | *PRMT3* | intron variant | 5.202 | 3.50E-06 |
| 11 | rs6483678 | 20416560 | C | T | *PRMT3* | intron variant | 5.202 | 3.50E-06 |
| 11 | rs1814181 | 20441566 | T | C | *PRMT3* | intron variant | 5.202 | 3.50E-06 |
| 11 | rs1596377 | 20432625 | A | G | *PRMT3* | intron variant | 5.202 | 3.50E-06 |
| 11 | rs1596375 | 20432832 | T | C | *PRMT3* | intron variant | 5.202 | 3.50E-06 |
| 11 | rs10833324 | 20435394 | A | G | *PRMT3* | intron variant | 5.202 | 3.50E-06 |
| 11 | rs10766681 | 20504688 | G | A | *PRMT3* | intron variant | 5.202 | 3.50E-06 |
| 11 | rs10766674 | 20430745 | G | C | *PRMT3* | intron variant | 5.202 | 3.50E-06 |
| 11 | rs10766673 | 20421095 | G | C | *PRMT3* | intron variant | 5.202 | 3.50E-06 |
| 11 | rs10766672 | 20417162 | C | T | *PRMT3* | intron variant | 5.202 | 3.50E-06 |
| 11 | rs1348402 | 20506232 | T | A | *PRMT3* | intron variant | 5.203 | 3.68E-06 |
| 11 | rs10741835 | 20398186 | G | A | *PRMT3* | intron variant | 5.304 | 3.70E-06 |
| 11 | rs10741839 | 20431376 | C | G | *PRMT3* | intron variant | 5.197 | 3.73E-06 |
| 11 | rs1561993 | 20510106 | T | C | *PRMT3* | downstream gene variant | 5.215 | 3.82E-06 |
| 11 | rs7101539 | 20444451 | C | T | *PRMT3* | intron variant | 5.204 | 3.86E-06 |
| 11 | rs10766666 | 20391371 | G | A | *PRMT3* | upstream gene variant | 5.241 | 4.11E-06 |
| 11 | rs7945941 | 20386358 | G | C | *PRMT3* | upstream gene variant | 5.137 | 4.29E-06 |
| 11 | rs7943077 | 20386184 | A | G | *PRMT3* | upstream gene variant | 5.137 | 4.29E-06 |
| 11 | rs4757947 | 20384196 | T | C | *PRMT3* | upstream gene variant | 5.137 | 4.29E-06 |
| 11 | rs2403590 | 20384100 | G | T | *PRMT3* | upstream gene variant | 5.137 | 4.29E-06 |
| 11 | rs2403589 | 20383939 | T | C | *PRMT3* | upstream gene variant | 5.137 | 4.29E-06 |
| 11 | rs2403588 | 20383935 | G | A | *PRMT3* | upstream gene variant | 5.137 | 4.29E-06 |
| 11 | rs11025541 | 20386711 | T | C | *PRMT3* | upstream gene variant | 5.137 | 4.29E-06 |
| 11 | rs11025539 | 20386510 | A | T | *PRMT3* | upstream gene variant | 5.137 | 4.29E-06 |
| 11 | rs11025538 | 20386161 | C | G | *PRMT3* | upstream gene variant | 5.137 | 4.29E-06 |
| 11 | rs10833316 | 20385650 | C | T | *PRMT3* | upstream gene variant | 5.137 | 4.29E-06 |
| 11 | rs7937901 | 20502447 | G | A | *PRMT3* | intron variant | 5.128 | 4.47E-06 |
| 11 | rs7935928 | 20496949 | C | G | *PRMT3* | intron variant | 5.128 | 4.47E-06 |
| 11 | rs6483674 | 20402591 | G | A | *PRMT3* | intron variant | 5.18 | 5.06E-06 |
| 11 | rs79308589 | 20386647 | G | T | *PRMT3* | upstream gene variant | 4.964 | 8.84E-06 |
| 9 | rs78537096 | 109604871 | C | T | *PALM2AKAP2* | intron variant | 24.42 | 6.97E-06 |
| 11 | rs2055246 | 20381340 | T | C | *HTATIP2* | intron variant | 5.512 | 2.55E-06 |
| 11 | rs7934001 | 20380289 | C | T | *HTATIP2* | intron variant | 5.397 | 3.50E-06 |
| 11 | rs3824886 | 20383067 | G | T | *HTATIP2* | missense variant | 5.137 | 4.29E-06 |
| 11 | rs3781679 | 20383211 | C | T | *HTATIP2* | 3 prime UTR variant | 5.137 | 4.29E-06 |
| 11 | rs2403587 | 20382960 | C | T | *HTATIP2* | intron variant | 5.137 | 4.29E-06 |
| 11 | rs7936865 | 20380394 | C | T | *HTATIP2* | intron variant | 5.199 | 4.96E-06 |
| 11 | rs2055244 | 20381156 | C | T | *HTATIP2* | intron variant | 5.199 | 4.96E-06 |
| 11 | rs2403586 | 20382708 | T | A | *HTATIP2* | intron variant | 5.193 | 5.60E-06 |
| 3 | rs73809055 | 7291173 | G | C | *GRM7* | intron variant | 18.93 | 7.90E-06 |
| 3 | rs57521140 | 7282362 | G | C | *GRM7* | intron variant | 18.93 | 7.90E-06 |
| 3 | rs141134664 | 7281556 | T | C | *GRM7* | intron variant | 18.93 | 7.90E-06 |
| 8 | rs11993294 | 25181608 | A | G | *DOCK5* | upstream gene variant | 17.85 | 2.80E-06 |

CHR chromosome, SNP single-nucleotide polymorphism, A1 Minor allele, A2 Major allele.

Supplementary table S3. The eQTL analysis results for genome-wide significant SNPs from the published human regions *cis*-eQTL database.

| SNP | Position | Gene | aveALL | CRBL | FCTX | HIPP | MEDU | OCTX | PUTM | SNIG | TCTX | THAL | WHMT |
| --- | --- | --- | --- | --- | --- | --- | --- | --- | --- | --- | --- | --- | --- |
| rs2055244 | chr11:20402702 | DBX1 | 2.00E-01 | 1.40E-01 | 8.00E-02 | 5.00E-01 | 9.50E-01 | 4.40E-01 | 9.70E-02 | 3.80E-01 | 2.80E-01 | 6.90E-  01 | 3.20E-01 |
|  | chr11:20402702 | FLJ13439 | 8.20E-01 | 8.60E-01 | 7.90E-01 | 1.60E-01 | 3.80E-01 | 2.00E-01 | 2.80E-01 | 9.70E-01 | 9.90E-01 | 3.20E-02 | 5.70E-01 |
|  | chr11:20402702 | FLJ13439 | 3.80E-01 | 8.40E-01 | 6.90E-01 | 5.90E-01 | 5.20E-01 | 3.70E-02 | 4.90E-01 | 3.00E-01 | 5.20E-01 | 7.10E-01 | 1.80E-01 |
|  | chr11:20402702 | FLJ13439 | 1.30E-01 | 2.40E-01 | 3.40E-01 | 2.20E-01 | 1.40E-01 | 8.10E-01 | 5.50E-02 | 7.10E-01 | 5.00E-01 | 6.20E-02 | 7.00E-01 |
|  | chr11:20402702 | FLJ13439 | 5.30E-01 | 8.80E-01 | 5.00E-01 | 6.10E-01 | 3.90E-01 | 7.40E-02 | 7.30E-01 | 2.60E-01 | 3.20E-01 | 7.40E-02 | 9.50E-02 |
|  | chr11:20402702 | FLJ13439 | 1.40E-01 | 6.40E-01 | 8.60E-01 | 2.40E-01 | 9.00E-01 | 1.70E-01 | 5.40E-01 | 9.80E-01 | 3.10E-01 | 2.50E-01 | 1.30E-01 |
|  | chr11:20402702 | HTATIP2 | 8.20E-01 | 9.90E-01 | 1.10E-02 | 8.60E-01 | 2.40E-01 | 5.70E-01 | 8.20E-01 | 7.00E-01 | 1.90E-01 | 4.70E-01 | 5.50E-01 |
|  | chr11:20402702 | HTATIP2 | 5.90E-01 | 3.30E-01 | 4.10E-01 | 5.20E-01 | 7.90E-01 | 3.30E-01 | 2.70E-01 | 4.80E-01 | 1.60E-02 | 5.80E-01 | 5.50E-01 |
|  | chr11:20402702 | HTATIP2 | 4.80E-01 | 7.40E-01 | 2.00E-01 | 7.10E-01 | 4.50E-02 | 3.00E-02 | 7.00E-01 | 3.80E-01 | 7.50E-01 | 5.00E-  01 | 4.20E-01 |
|  | chr11:20402886 | DBX1 | 7.70E-02 | 1.40E-04 | 3.30E-02 | 6.10E-01 | 5.10E-01 | 3.10E-01 | 6.90E-01 | 5.90E-01 | 4.30E-01 | 7.40E-  01 | 6.40E-01 |
|  | chr11:20402886 | DBX1 | 7.90E-01 | 1.60E-02 | 4.70E-01 | 3.70E-01 | 9.70E-01 | 8.40E-01 | 9.10E-01 | 3.70E-01 | 2.70E-02 | 7.60E-  01 | 7.40E-01 |
|  | chr11:20402886 | DBX1 | 1.50E-01 | 7.90E-02 | 7.30E-01 | 2.50E-02 | 1.90E-01 | 3.60E-01 | 6.20E-01 | 3.30E-01 | 9.30E-01 | 6.00E-  01 | 8.20E-01 |
|  | chr11:20402886 | DBX1 | 1.50E-01 | 1.60E-01 | 3.60E-02 | 3.70E-01 | 9.70E-01 | 5.60E-01 | 1.50E-01 | 3.20E-01 | 3.50E-01 | 7.20E-  01 | 2.80E-01 |
|  | chr11:20402886 | DBX1 | 3.60E-01 | 5.90E-02 | 9.60E-01 | 6.10E-01 | 5.00E-01 | 8.40E-01 | 8.50E-01 | 8.70E-01 | 5.60E-01 | 8.00E-  01 | 5.30E-01 |
| rs2055246 | chr11:20402886 | DBX1 | 6.10E-01 | 3.70E-01 | 9.40E-02 | 4.40E-01 | 7.50E-01 | 4.00E-01 | 4.60E-01 | 4.10E-01 | 6.50E-01 | 2.00E-  01 | 5.00E-01 |
|  | chr11:20402886 | FLJ13439 | 3.70E-01 | 9.30E-01 | 6.60E-01 | 5.60E-01 | 5.20E-01 | 2.20E-02 | 4.20E-01 | 3.40E-01 | 4.80E-01 | 7.00E-  01 | 2.00E-01 |
|  | chr11:20402886 | FLJ13439 | 8.60E-01 | 8.70E-01 | 7.50E-01 | 1.90E-01 | 5.00E-01 | 2.00E-01 | 2.50E-01 | 9.50E-01 | 1.00E+00 | 2.60E-  02 | 4.70E-01 |
|  | chr11:20402886 | FLJ13439 | 1.50E-01 | 3.00E-01 | 4.00E-01 | 2.70E-01 | 1.40E-01 | 8.30E-01 | 9.00E-02 | 7.50E-01 | 4.90E-01 | 4.90E-  02 | 7.30E-01 |
|  | chr11:20402886 | FLJ13439 | 5.30E-01 | 8.90E-01 | 4.60E-01 | 7.10E-01 | 4.50E-01 | 9.90E-02 | 6.80E-01 | 3.60E-01 | 3.20E-01 | 9.30E-  02 | 5.00E-02 |
| rs2403586 | chr11:20404254 | DBX1 | 1.90E-01 | 1.40E-01 | 8.00E-02 | 5.00E-01 | 9.50E-01 | 4.40E-01 | 9.70E-02 | 3.80E-01 | 2.80E-01 | 6.90E-  01 | 3.30E-01 |
|  | chr11:20404254 | FLJ13439 | 8.10E-01 | 8.60E-01 | 7.90E-01 | 1.60E-01 | 3.80E-01 | 2.00E-01 | 2.80E-01 | 9.60E-01 | 9.90E-01 | 3.20E-  02 | 5.70E-01 |
|  | chr11:20404254 | FLJ13439 | 3.80E-01 | 8.40E-01 | 6.80E-01 | 5.90E-01 | 5.20E-01 | 3.70E-02 | 4.90E-01 | 3.00E-01 | 5.20E-01 | 7.10E-  01 | 1.80E-01 |
|  | chr11:20404254 | FLJ13439 | 1.30E-01 | 2.40E-01 | 3.40E-01 | 2.10E-01 | 1.40E-01 | 8.10E-01 | 5.60E-02 | 7.20E-01 | 5.00E-01 | 6.10E-  02 | 7.00E-01 |
|  | chr11:20404254 | FLJ13439 | 5.20E-01 | 8.80E-01 | 5.00E-01 | 6.10E-01 | 4.00E-01 | 7.40E-02 | 7.30E-01 | 2.60E-01 | 3.20E-01 | 7.20E-  02 | 9.50E-02 |
|  | chr11:20404254 | FLJ13439 | 1.40E-01 | 6.40E-01 | 8.60E-01 | 2.40E-01 | 9.00E-01 | 1.70E-01 | 5.30E-01 | 9.90E-01 | 3.10E-01 | 2.50E-  01 | 1.30E-01 |
|  | chr11:20404254 | HTATIP2 | 8.20E-01 | 9.90E-01 | 1.10E-02 | 8.60E-01 | 2.40E-01 | 5.70E-01 | 8.20E-01 | 7.10E-01 | 1.90E-01 | 4.70E-  01 | 5.60E-01 |
|  | chr11:20404254 | HTATIP2 | 5.90E-01 | 3.30E-01 | 4.20E-01 | 5.20E-01 | 8.00E-01 | 3.40E-01 | 2.80E-01 | 4.80E-01 | 1.60E-02 | 5.90E-  01 | 5.50E-01 |
|  | chr11:20404254 | HTATIP2 | 4.90E-01 | 7.30E-01 | 2.00E-01 | 7.00E-01 | 4.50E-02 | 2.90E-02 | 7.00E-01 | 3.80E-01 | 7.50E-01 | 5.00E-  01 | 4.30E-01 |
|  | chr11:20404254 | HTATIP2 | 8.70E-02 | 6.70E-01 | 9.90E-01 | 8.40E-02 | 9.20E-02 | 4.80E-02 | 7.60E-01 | 3.20E-01 | 7.60E-01 | 7.10E-  01 | 3.20E-01 |
|  | chr11:20405481 | DBX1 | 8.50E-02 | 9.70E-05 | 3.70E-02 | 6.80E-01 | 5.70E-01 | 3.00E-01 | 5.60E-01 | 6.20E-01 | 5.00E-01 | 7.40E-  01 | 6.20E-01 |
| rs2403588 | chr11:20405481 | DBX1 | 6.80E-01 | 1.10E-02 | 4.90E-01 | 5.00E-01 | 8.90E-01 | 8.90E-01 | 9.50E-01 | 3.80E-01 | 2.70E-02 | 6.70E-  01 | 6.30E-01 |
|  | chr11:20405481 | DBX1 | 1.50E-01 | 7.80E-02 | 6.10E-01 | 4.00E-02 | 2.90E-01 | 4.60E-01 | 6.60E-01 | 3.50E-01 | 8.80E-01 | 4.50E-  01 | 8.00E-01 |
|  | chr11:20405481 | DBX1 | 3.30E-01 | 5.50E-02 | 9.90E-01 | 6.80E-01 | 4.40E-01 | 9.30E-01 | 8.50E-01 | 8.80E-01 | 6.60E-01 | 8.10E-  01 | 5.00E-01 |
|  | chr11:20405481 | DBX1 | 6.80E-01 | 4.60E-01 | 7.20E-02 | 4.20E-01 | 9.00E-01 | 3.00E-01 | 5.50E-01 | 4.90E-01 | 5.40E-01 | 1.30E-  01 | 4.80E-01 |
|  | chr11:20405481 | DBX1 | 2.00E-01 | 1.40E-01 | 8.00E-02 | 5.00E-01 | 9.50E-01 | 4.40E-01 | 9.70E-02 | 3.80E-01 | 2.90E-01 | 6.90E-  01 | 3.20E-01 |
|  | chr11:20405481 | FLJ13439 | 8.20E-01 | 8.60E-01 | 7.90E-01 | 1.60E-01 | 3.90E-01 | 2.00E-01 | 2.80E-01 | 9.70E-01 | 9.90E-01 | 3.20E-  02 | 5.70E-01 |
|  | chr11:20405481 | FLJ13439 | 3.80E-01 | 8.40E-01 | 6.90E-01 | 5.90E-01 | 5.20E-01 | 3.70E-02 | 4.90E-01 | 3.00E-01 | 5.20E-01 | 7.10E-  01 | 1.80E-01 |
|  | chr11:20405481 | FLJ13439 | 1.30E-01 | 2.40E-01 | 3.40E-01 | 2.20E-01 | 1.40E-01 | 8.10E-01 | 5.60E-02 | 7.10E-01 | 5.00E-01 | 6.20E-  02 | 7.00E-01 |
|  | chr11:20405481 | FLJ13439 | 5.30E-01 | 8.80E-01 | 5.00E-01 | 6.10E-01 | 4.00E-01 | 7.40E-02 | 7.30E-01 | 2.60E-01 | 3.20E-01 | 7.30E-  02 | 9.50E-02 |
|  | chr11:20405646 | DBX1 | 8.50E-02 | 9.70E-05 | 3.70E-02 | 6.80E-01 | 5.70E-01 | 3.00E-01 | 5.60E-01 | 6.20E-01 | 5.00E-01 | 7.40E-  01 | 6.20E-01 |
|  | chr11:20405646 | DBX1 | 6.80E-01 | 1.10E-02 | 4.90E-01 | 5.00E-01 | 8.90E-01 | 8.90E-01 | 9.50E-01 | 3.80E-01 | 2.70E-02 | 6.70E-  01 | 6.30E-01 |
|  | chr11:20405646 | DBX1 | 1.50E-01 | 7.80E-02 | 6.10E-01 | 4.00E-02 | 2.90E-01 | 4.60E-01 | 6.60E-01 | 3.50E-01 | 8.80E-01 | 4.50E-  01 | 8.00E-01 |
|  | chr11:20405646 | DBX1 | 3.30E-01 | 5.50E-02 | 1.00E+00 | 6.80E-01 | 4.40E-01 | 9.30E-01 | 8.50E-01 | 8.80E-01 | 6.60E-01 | 8.10E-  01 | 5.00E-01 |
|  | chr11:20405646 | DBX1 | 6.80E-01 | 4.60E-01 | 7.20E-02 | 4.20E-01 | 9.00E-01 | 3.00E-01 | 5.50E-01 | 4.90E-01 | 5.40E-01 | 1.30E-  01 | 4.80E-01 |
|  | chr11:20405646 | DBX1 | 2.00E-01 | 1.40E-01 | 8.00E-02 | 5.00E-01 | 9.50E-01 | 4.40E-01 | 9.70E-02 | 3.80E-01 | 2.90E-01 | 6.90E-  01 | 3.20E-01 |
|  | chr11:20405646 | FLJ13439 | 8.20E-01 | 8.60E-01 | 7.90E-01 | 1.60E-01 | 3.90E-01 | 2.00E-01 | 2.80E-01 | 9.70E-01 | 9.90E-01 | 3.20E-  02 | 5.70E-01 |
| rs2403590 | chr11:20405646 | FLJ13439 | 3.80E-01 | 8.40E-01 | 6.90E-01 | 5.90E-01 | 5.20E-01 | 3.70E-02 | 4.90E-01 | 3.00E-01 | 5.20E-01 | 7.10E-  01 | 1.80E-01 |
|  | chr11:20405646 | FLJ13439 | 1.30E-01 | 2.40E-01 | 3.40E-01 | 2.20E-01 | 1.40E-01 | 8.10E-01 | 5.60E-02 | 7.10E-01 | 5.00E-01 | 6.20E-  02 | 7.00E-01 |
|  | chr11:20405646 | FLJ13439 | 5.30E-01 | 8.80E-01 | 5.00E-01 | 6.10E-01 | 4.00E-01 | 7.40E-02 | 7.30E-01 | 2.60E-01 | 3.20E-01 | 7.30E-  02 | 9.50E-02 |
| rs3781679 | chr11:20404757 | DBX1 | 2.00E-01 | 1.40E-01 | 8.00E-02 | 5.00E-01 | 9.50E-01 | 4.40E-01 | 9.70E-02 | 3.80E-01 | 2.90E-01 | 6.90E-  01 | 3.20E-01 |
|  | chr11:20404757 | FLJ13439 | 8.20E-01 | 8.60E-01 | 7.90E-01 | 1.60E-01 | 3.90E-01 | 2.00E-01 | 2.80E-01 | 9.70E-01 | 9.90E-01 | 3.20E-  02 | 5.70E-01 |
|  | chr11:20404757 | FLJ13439 | 3.80E-01 | 8.40E-01 | 6.90E-01 | 5.90E-01 | 5.20E-01 | 3.70E-02 | 4.90E-01 | 3.00E-01 | 5.20E-01 | 7.10E-  01 | 1.80E-01 |
|  | chr11:20404757 | FLJ13439 | 1.30E-01 | 2.40E-01 | 3.40E-01 | 2.20E-01 | 1.40E-01 | 8.10E-01 | 5.60E-02 | 7.10E-01 | 5.00E-01 | 6.20E-  02 | 7.00E-01 |
|  | chr11:20404757 | FLJ13439 | 5.30E-01 | 8.80E-01 | 5.00E-01 | 6.10E-01 | 4.00E-01 | 7.40E-02 | 7.30E-01 | 2.60E-01 | 3.20E-01 | 7.30E-  02 | 9.50E-02 |
|  | chr11:20404757 | FLJ13439 | 1.40E-01 | 6.40E-01 | 8.60E-01 | 2.40E-01 | 9.00E-01 | 1.70E-01 | 5.30E-01 | 9.90E-01 | 3.10E-01 | 2.50E-  01 | 1.30E-01 |
|  | chr11:20404757 | HTATIP2 | 8.20E-01 | 9.90E-01 | 1.10E-02 | 8.60E-01 | 2.40E-01 | 5.70E-01 | 8.20E-01 | 7.00E-01 | 1.90E-01 | 4.80E-  01 | 5.50E-01 |
|  | chr11:20404757 | HTATIP2 | 5.80E-01 | 3.30E-01 | 4.10E-01 | 5.20E-01 | 7.90E-01 | 3.30E-01 | 2.80E-01 | 4.80E-01 | 1.60E-02 | 5.80E-  01 | 5.50E-01 |
|  | chr11:20404757 | HTATIP2 | 4.80E-01 | 7.30E-01 | 2.00E-01 | 7.10E-01 | 4.50E-02 | 3.00E-02 | 7.00E-01 | 3.80E-01 | 7.50E-01 | 5.00E-  01 | 4.20E-01 |
|  | chr11:20404613 | DBX1 | 8.50E-02 | 1.10E-04 | 3.40E-02 | 6.80E-01 | 5.70E-01 | 2.90E-01 | 5.60E-01 | 6.60E-01 | 4.80E-01 | 7.50E-  01 | 6.40E-01 |
|  | chr11:20404613 | DBX1 | 6.80E-01 | 1.10E-02 | 4.80E-01 | 5.00E-01 | 8.80E-01 | 8.90E-01 | 9.20E-01 | 3.90E-01 | 2.50E-02 | 6.60E-  01 | 6.40E-01 |
|  | chr11:20404613 | DBX1 | 1.50E-01 | 8.50E-02 | 6.10E-01 | 4.00E-02 | 2.70E-01 | 4.40E-01 | 6.80E-01 | 3.60E-01 | 8.90E-01 | 4.50E-  01 | 7.70E-01 |
|  | chr11:20404613 | DBX1 | 3.10E-01 | 5.80E-02 | 9.90E-01 | 6.90E-01 | 4.70E-01 | 9.20E-01 | 8.50E-01 | 9.20E-01 | 7.20E-01 | 8.00E-  01 | 5.00E-01 |
| rs3824886 | chr11:20404613 | DBX1 | 6.90E-01 | 4.50E-01 | 7.00E-02 | 4.30E-01 | 8.90E-01 | 2.90E-01 | 5.90E-01 | 5.00E-01 | 5.30E-01 | 1.30E-  01 | 5.10E-01 |
|  | chr11:20404613 | DBX1 | 2.00E-01 | 1.40E-01 | 7.60E-02 | 5.30E-01 | 9.60E-01 | 4.60E-01 | 9.70E-02 | 3.70E-01 | 3.00E-01 | 6.80E-  01 | 3.10E-01 |
|  | chr11:20404613 | FLJ13439 | 8.80E-01 | 8.20E-01 | 7.80E-01 | 1.90E-01 | 4.10E-01 | 1.90E-01 | 2.90E-01 | 9.60E-01 | 9.60E-01 | 3.30E-  02 | 6.00E-01 |
|  | chr11:20404613 | FLJ13439 | 4.30E-01 | 8.20E-01 | 6.80E-01 | 5.50E-01 | 5.00E-01 | 3.80E-02 | 4.70E-01 | 3.00E-01 | 4.90E-01 | 7.60E-  01 | 1.90E-01 |
|  | chr11:20404613 | FLJ13439 | 1.30E-01 | 2.50E-01 | 3.40E-01 | 2.10E-01 | 1.20E-01 | 7.90E-01 | 6.20E-02 | 7.20E-01 | 4.90E-01 | 6.30E-  02 | 6.50E-01 |
| rs4757947 | chr11:20405742 | DBX1 | 8.50E-02 | 9.70E-05 | 3.70E-02 | 6.80E-01 | 5.70E-01 | 3.00E-01 | 5.60E-01 | 6.20E-01 | 5.00E-01 | 7.40E-  01 | 6.20E-01 |
|  | chr11:20405742 | DBX1 | 6.80E-01 | 1.10E-02 | 4.90E-01 | 5.00E-01 | 8.90E-01 | 8.90E-01 | 9.50E-01 | 3.80E-01 | 2.70E-02 | 6.70E-  01 | 6.30E-01 |
|  | chr11:20405742 | DBX1 | 1.50E-01 | 7.80E-02 | 6.10E-01 | 4.00E-02 | 2.90E-01 | 4.60E-01 | 6.60E-01 | 3.50E-01 | 8.80E-01 | 4.50E-  01 | 8.00E-01 |
|  | chr11:20405742 | DBX1 | 3.30E-01 | 5.50E-02 | 1.00E+00 | 6.80E-01 | 4.40E-01 | 9.30E-01 | 8.50E-01 | 8.80E-01 | 6.60E-01 | 8.10E-  01 | 5.00E-01 |
|  | chr11:20405742 | DBX1 | 6.80E-01 | 4.60E-01 | 7.20E-02 | 4.20E-01 | 9.00E-01 | 3.00E-01 | 5.50E-01 | 4.90E-01 | 5.40E-01 | 1.30E-  01 | 4.80E-01 |
|  | chr11:20405742 | DBX1 | 2.00E-01 | 1.40E-01 | 8.00E-02 | 5.00E-01 | 9.50E-01 | 4.40E-01 | 9.70E-02 | 3.80E-01 | 2.90E-01 | 6.90E-  01 | 3.20E-01 |
|  | chr11:20405742 | FLJ13439 | 8.20E-01 | 8.60E-01 | 7.90E-01 | 1.60E-01 | 3.90E-01 | 2.00E-01 | 2.80E-01 | 9.70E-01 | 9.90E-01 | 3.20E-  02 | 5.70E-01 |
|  | chr11:20405742 | FLJ13439 | 3.80E-01 | 8.40E-01 | 6.90E-01 | 5.90E-01 | 5.20E-01 | 3.70E-02 | 4.90E-01 | 3.00E-01 | 5.20E-01 | 7.10E-  01 | 1.80E-01 |
|  | chr11:20405742 | FLJ13439 | 1.30E-01 | 2.40E-01 | 3.40E-01 | 2.20E-01 | 1.40E-01 | 8.10E-01 | 5.60E-02 | 7.10E-01 | 5.00E-01 | 6.20E-  02 | 7.00E-01 |
|  | chr11:20405742 | FLJ13439 | 5.30E-01 | 8.80E-01 | 5.00E-01 | 6.10E-01 | 4.00E-01 | 7.40E-02 | 7.30E-01 | 2.60E-01 | 3.20E-01 | 7.30E-  02 | 9.50E-02 |
|  | chr11:20401835 | DBX1 | 2.20E-01 | 1.50E-01 | 8.40E-02 | 5.00E-01 | 9.80E-01 | 4.80E-01 | 8.80E-02 | 3.80E-01 | 3.20E-01 | 6.70E-  01 | 3.00E-01 |
| rs7934001 | chr11:20401835 | DBX1 | 6.00E-01 | 3.80E-01 | 8.50E-02 | 4.00E-01 | 7.40E-01 | 2.80E-01 | 5.40E-01 | 5.10E-01 | 5.90E-01 | 1.70E-  01 | 5.00E-01 |
|  | chr11:20401835 | FLJ13439 | 3.40E-01 | 8.20E-01 | 6.50E-01 | 7.10E-01 | 5.50E-01 | 2.80E-02 | 4.40E-01 | 3.10E-01 | 4.90E-01 | 6.90E-  01 | 1.80E-01 |
|  | chr11:20401835 | FLJ13439 | 8.00E-01 | 8.30E-01 | 7.50E-01 | 1.80E-01 | 4.00E-01 | 1.90E-01 | 2.40E-01 | 9.70E-01 | 9.30E-01 | 3.10E-  02 | 5.50E-01 |
|  | chr11:20401835 | FLJ13439 | 5.70E-01 | 8.50E-01 | 4.50E-01 | 6.50E-01 | 3.80E-01 | 1.00E-01 | 6.60E-01 | 2.70E-01 | 3.30E-01 | 8.10E-  02 | 5.40E-02 |
|  | chr11:20401835 | FLJ13439 | 1.50E-01 | 2.70E-01 | 4.30E-01 | 1.80E-01 | 1.70E-01 | 7.50E-01 | 7.80E-02 | 7.10E-01 | 4.60E-01 | 6.80E-  02 | 6.50E-01 |
|  | chr11:20401835 | FLJ13439 | 1.50E-01 | 6.80E-01 | 9.50E-01 | 2.20E-01 | 9.30E-01 | 1.60E-01 | 6.10E-01 | 9.80E-01 | 3.10E-01 | 2.70E-  01 | 1.10E-01 |
|  | chr11:20401835 | HTATIP2 | 8.80E-01 | 9.50E-01 | 1.70E-02 | 8.30E-01 | 2.10E-01 | 5.60E-01 | 8.30E-01 | 7.10E-01 | 1.90E-01 | 5.10E-  01 | 4.70E-01 |
|  | chr11:20401835 | HTATIP2 | 5.80E-01 | 3.50E-01 | 4.50E-01 | 5.80E-01 | 8.40E-01 | 3.40E-01 | 3.20E-01 | 4.80E-01 | 2.30E-02 | 5.90E-  01 | 5.00E-01 |
|  | chr11:20401835 | HTATIP2 | 4.30E-01 | 8.10E-01 | 2.30E-01 | 6.80E-01 | 3.80E-02 | 2.70E-02 | 7.90E-01 | 3.90E-01 | 7.60E-01 | 4.60E-  01 | 4.10E-01 |
|  | chr11:20401940 | DBX1 | 1.40E-01 | 8.00E-02 | 5.00E-01 | 9.50E-01 | 4.40E-01 | 9.70E-02 | 3.80E-01 | 2.80E-01 | 6.90E-01 | 3.20E-  01 | 1.40E-01 |
|  | chr11:20401940 | FLJ13439 | 8.60E-01 | 7.90E-01 | 1.60E-01 | 3.80E-01 | 2.00E-01 | 2.80E-01 | 9.70E-01 | 9.90E-01 | 3.20E-02 | 5.70E-  01 | 8.60E-01 |
|  | chr11:20401940 | FLJ13439 | 8.40E-01 | 6.90E-01 | 5.90E-01 | 5.20E-01 | 3.70E-02 | 4.90E-01 | 3.00E-01 | 5.20E-01 | 7.10E-01 | 1.80E-  01 | 8.40E-01 |
|  | chr11:20401940 | FLJ13439 | 2.40E-01 | 3.40E-01 | 2.20E-01 | 1.40E-01 | 8.10E-01 | 5.50E-02 | 7.10E-01 | 5.00E-01 | 6.20E-02 | 7.00E-  01 | 2.40E-01 |
|  | chr11:20401940 | FLJ13439 | 8.80E-01 | 5.00E-01 | 6.10E-01 | 3.90E-01 | 7.40E-02 | 7.30E-01 | 2.60E-01 | 3.20E-01 | 7.40E-02 | 9.50E-  02 | 8.80E-01 |
|  | chr11:20401940 | FLJ13439 | 6.40E-01 | 8.60E-01 | 2.40E-01 | 9.00E-01 | 1.70E-01 | 5.40E-01 | 9.80E-01 | 3.10E-01 | 2.50E-01 | 1.30E-  01 | 6.40E-01 |
|  | chr11:20401940 | HTATIP2 | 9.90E-01 | 1.10E-02 | 8.60E-01 | 2.40E-01 | 5.70E-01 | 8.20E-01 | 7.00E-01 | 1.90E-01 | 4.70E-01 | 5.50E-  01 | 9.90E-01 |
| rs7936865 | chr11:20401940 | HTATIP2 | 3.30E-01 | 4.10E-01 | 5.20E-01 | 7.90E-01 | 3.30E-01 | 2.70E-01 | 4.80E-01 | 1.60E-02 | 5.80E-01 | 5.50E-  01 | 3.30E-01 |
|  | chr11:20401940 | HTATIP2 | 7.40E-01 | 2.00E-01 | 7.10E-01 | 4.50E-02 | 3.00E-02 | 7.00E-01 | 3.80E-01 | 7.50E-01 | 5.00E-01 | 4.20E-  01 | 7.40E-01 |
|  | chr11:20401940 | HTATIP2 | 6.70E-01 | 9.90E-01 | 8.40E-02 | 9.30E-02 | 4.90E-02 | 7.60E-01 | 3.20E-01 | 7.60E-01 | 7.10E-01 | 3.20E-  01 | 6.70E-01 |
| rs7943077 | chr11:20407730 | DBX1 | 9.70E-02 | 1.80E-04 | 9.00E-02 | 6.70E-01 | 6.80E-01 | 1.40E-01 | 4.40E-01 | 9.10E-01 | 5.30E-01 | 8.20E-  01 | 8.50E-01 |
|  | chr11:20407730 | DBX1 | 7.00E-01 | 1.90E-02 | 5.90E-01 | 5.60E-01 | 8.90E-01 | 7.80E-01 | 5.40E-01 | 3.20E-01 | 2.70E-02 | 4.90E-  01 | 4.30E-01 |
|  | chr11:20407730 | DBX1 | 2.20E-01 | 6.20E-02 | 9.20E-01 | 9.10E-01 | 4.60E-01 | 6.80E-01 | 9.30E-01 | 5.00E-01 | 9.80E-01 | 8.70E-  01 | 4.80E-01 |
|  | chr11:20407730 | DBX1 | 2.20E-01 | 7.70E-02 | 9.20E-01 | 7.40E-02 | 3.50E-01 | 2.40E-01 | 4.90E-01 | 5.70E-01 | 6.70E-01 | 4.40E-  01 | 4.00E-01 |
|  | chr11:20407730 | DBX1 | 2.10E-01 | 1.30E-01 | 8.50E-02 | 5.50E-01 | 8.10E-01 | 2.50E-01 | 1.80E-01 | 2.70E-01 | 4.80E-01 | 6.80E-  01 | 1.50E-01 |
|  | chr11:20407730 | DBX1 | 9.00E-01 | 3.60E-01 | 1.80E-01 | 7.30E-01 | 9.90E-01 | 3.40E-01 | 9.30E-01 | 4.40E-01 | 4.10E-01 | 2.00E-  01 | 7.60E-01 |
|  | chr11:20407730 | FLJ13439 | 8.90E-01 | 6.10E-01 | 6.20E-01 | 3.90E-01 | 6.60E-01 | 2.20E-01 | 2.80E-01 | 6.70E-01 | 8.60E-01 | 1.50E-  02 | 9.40E-01 |
|  | chr11:20407730 | FLJ13439 | 8.20E-02 | 1.80E-01 | 5.10E-01 | 6.70E-02 | 8.30E-02 | 5.90E-01 | 7.90E-02 | 9.70E-01 | 5.30E-01 | 7.60E-  02 | 6.60E-01 |
|  | chr11:20407730 | FLJ13439 | 5.00E-01 | 8.30E-01 | 4.30E-01 | 7.60E-01 | 7.10E-01 | 2.00E-01 | 6.80E-01 | 2.70E-01 | 3.50E-01 | 9.20E-  02 | 6.90E-02 |
|  | chr11:20407730 | FLJ13439 | 5.50E-01 | 5.70E-01 | 6.90E-01 | 5.80E-01 | 3.00E-01 | 1.40E-01 | 4.60E-01 | 1.60E-01 | 3.70E-01 | 7.90E-  01 | 1.70E-01 |
|  | chr11:20407904 | DBX1 | 9.70E-02 | 1.80E-04 | 9.00E-02 | 6.70E-01 | 6.80E-01 | 1.40E-01 | 4.40E-01 | 9.10E-01 | 5.30E-01 | 8.20E-01 | 8.50E-  01 |
|  | chr11:20407904 | DBX1 | 7.00E-01 | 1.90E-02 | 5.90E-01 | 5.60E-01 | 8.90E-01 | 7.80E-01 | 5.40E-01 | 3.20E-01 | 2.70E-02 | 4.90E-01 | 4.30E-  01 |
|  | chr11:20407904 | DBX1 | 2.20E-01 | 6.20E-02 | 9.20E-01 | 9.10E-01 | 4.60E-01 | 6.80E-01 | 9.30E-01 | 5.00E-01 | 9.80E-01 | 8.70E-01 | 4.80E-  01 |
| rs7945941 | chr11:20407904 | DBX1 | 2.20E-01 | 7.70E-02 | 9.20E-01 | 7.40E-02 | 3.50E-01 | 2.40E-01 | 4.90E-01 | 5.70E-01 | 6.70E-01 | 4.40E-01 | 4.00E-  01 |
|  | chr11:20407904 | DBX1 | 2.10E-01 | 1.30E-01 | 8.50E-02 | 5.50E-01 | 8.10E-01 | 2.50E-01 | 1.80E-01 | 2.70E-01 | 4.80E-01 | 6.80E-01 | 1.50E-  01 |
|  | chr11:20407904 | DBX1 | 9.00E-01 | 3.60E-01 | 1.80E-01 | 7.30E-01 | 9.90E-01 | 3.40E-01 | 9.30E-01 | 4.40E-01 | 4.10E-01 | 2.00E-01 | 7.60E-  01 |
|  | chr11:20407904 | FLJ13439 | 8.90E-01 | 6.10E-01 | 6.20E-01 | 3.90E-01 | 6.60E-01 | 2.20E-01 | 2.80E-01 | 6.70E-01 | 8.60E-01 | 1.50E-02 | 9.40E-  01 |
|  | chr11:20407904 | FLJ13439 | 8.20E-02 | 1.80E-01 | 5.10E-01 | 6.60E-02 | 8.30E-02 | 5.90E-01 | 7.90E-02 | 9.70E-01 | 5.30E-01 | 7.60E-02 | 6.60E-  01 |
|  | chr11:20407904 | FLJ13439 | 5.00E-01 | 8.30E-01 | 4.30E-01 | 7.60E-01 | 7.10E-01 | 2.10E-01 | 6.80E-01 | 2.70E-01 | 3.50E-01 | 9.20E-02 | 6.90E-  02 |
|  | chr11:20407904 | FLJ13439 | 5.50E-01 | 5.70E-01 | 6.90E-01 | 5.80E-01 | 3.00E-01 | 1.40E-01 | 4.60E-01 | 1.60E-01 | 3.70E-01 | 7.90E-01 | 1.70E-  01 |
| rs10833316 | chr11:20407196 | DBX1 | 9.70E-02 | 1.80E-04 | 9.00E-02 | 6.70E-01 | 6.80E-01 | 1.40E-01 | 4.40E-01 | 9.10E-01 | 5.30E-01 | 8.20E-  01 | 8.50E-01 |
|  | chr11:20407196 | DBX1 | 7.00E-01 | 1.90E-02 | 5.90E-01 | 5.60E-01 | 8.90E-01 | 7.80E-01 | 5.40E-01 | 3.20E-01 | 2.70E-02 | 4.90E-  01 | 4.30E-01 |
|  | chr11:20407196 | DBX1 | 2.20E-01 | 6.20E-02 | 9.20E-01 | 9.10E-01 | 4.60E-01 | 6.80E-01 | 9.30E-01 | 5.00E-01 | 9.80E-01 | 8.70E-  01 | 4.80E-01 |
|  | chr11:20407196 | DBX1 | 2.20E-01 | 7.70E-02 | 9.20E-01 | 7.40E-02 | 3.50E-01 | 2.40E-01 | 4.90E-01 | 5.70E-01 | 6.70E-01 | 4.40E-  01 | 4.00E-01 |
|  | chr11:20407196 | DBX1 | 2.10E-01 | 1.30E-01 | 8.50E-02 | 5.50E-01 | 8.10E-01 | 2.50E-01 | 1.80E-01 | 2.70E-01 | 4.80E-01 | 6.80E-  01 | 1.50E-01 |
|  | chr11:20407196 | DBX1 | 9.00E-01 | 3.60E-01 | 1.80E-01 | 7.30E-01 | 9.90E-01 | 3.40E-01 | 9.30E-01 | 4.40E-01 | 4.10E-01 | 2.00E-  01 | 7.60E-01 |
|  | chr11:20407196 | FLJ13439 | 8.90E-01 | 6.20E-01 | 6.20E-01 | 3.90E-01 | 6.60E-01 | 2.20E-01 | 2.80E-01 | 6.70E-01 | 8.60E-01 | 1.50E-  02 | 9.40E-01 |
|  | chr11:20407196 | FLJ13439 | 8.20E-02 | 1.80E-01 | 5.10E-01 | 6.70E-02 | 8.30E-02 | 5.90E-01 | 7.90E-02 | 9.70E-01 | 5.30E-01 | 7.60E-  02 | 6.60E-01 |
|  | chr11:20407196 | FLJ13439 | 5.00E-01 | 8.30E-01 | 4.30E-01 | 7.60E-01 | 7.10E-01 | 2.00E-01 | 6.80E-01 | 2.70E-01 | 3.50E-01 | 9.20E-  02 | 6.90E-02 |
| rs11025538 | chr11:20407707 | DBX1 | 9.70E-02 | 1.80E-04 | 9.00E-02 | 6.70E-01 | 6.80E-01 | 1.40E-01 | 4.40E-01 | 9.10E-01 | 5.30E-01 | 8.20E-  01 | 8.50E-01 |
|  | chr11:20407707 | DBX1 | 7.00E-01 | 1.90E-02 | 5.90E-01 | 5.60E-01 | 8.90E-01 | 7.80E-01 | 5.40E-01 | 3.20E-01 | 2.70E-02 | 4.90E-  01 | 4.30E-01 |
|  | chr11:20407707 | DBX1 | 2.20E-01 | 6.20E-02 | 9.20E-01 | 9.10E-01 | 4.60E-01 | 6.80E-01 | 9.30E-01 | 5.00E-01 | 9.80E-01 | 8.70E-  01 | 4.80E-01 |
|  | chr11:20407707 | DBX1 | 2.20E-01 | 7.70E-02 | 9.20E-01 | 7.40E-02 | 3.50E-01 | 2.40E-01 | 4.90E-01 | 5.70E-01 | 6.70E-01 | 4.40E-  01 | 4.00E-01 |
|  | chr11:20407707 | DBX1 | 2.10E-01 | 1.30E-01 | 8.50E-02 | 5.50E-01 | 8.10E-01 | 2.50E-01 | 1.80E-01 | 2.70E-01 | 4.80E-01 | 6.80E-  01 | 1.50E-01 |
|  | chr11:20407707 | DBX1 | 9.00E-01 | 3.60E-01 | 1.80E-01 | 7.30E-01 | 9.90E-01 | 3.40E-01 | 9.30E-01 | 4.40E-01 | 4.10E-01 | 2.00E-  01 | 7.60E-01 |
|  | chr11:20407707 | FLJ13439 | 8.90E-01 | 6.20E-01 | 6.20E-01 | 3.90E-01 | 6.60E-01 | 2.20E-01 | 2.80E-01 | 6.70E-01 | 8.60E-01 | 1.50E-  02 | 9.40E-01 |
|  | chr11:20407707 | FLJ13439 | 8.20E-02 | 1.80E-01 | 5.10E-01 | 6.70E-02 | 8.30E-02 | 5.90E-01 | 7.90E-02 | 9.70E-01 | 5.30E-01 | 7.60E-  02 | 6.60E-01 |
|  | chr11:20407707 | FLJ13439 | 5.00E-01 | 8.30E-01 | 4.30E-01 | 7.60E-01 | 7.10E-01 | 2.00E-01 | 6.80E-01 | 2.70E-01 | 3.50E-01 | 9.20E-  02 | 6.90E-02 |
|  | chr11:20407707 | FLJ13439 | 5.50E-01 | 5.70E-01 | 6.90E-01 | 5.80E-01 | 3.00E-01 | 1.40E-01 | 4.60E-01 | 1.60E-01 | 3.70E-01 | 7.90E-  01 | 1.70E-01 |
|  | chr11:20408056 | DBX1 | 1.80E-04 | 9.00E-02 | 6.70E-01 | 6.90E-01 | 1.40E-01 | 4.40E-01 | 9.10E-01 | 5.30E-01 | 8.20E-01 | 8.50E-  01 | 1.80E-04 |
|  | chr11:20408056 | DBX1 | 1.90E-02 | 5.90E-01 | 5.60E-01 | 8.90E-01 | 7.80E-01 | 5.40E-01 | 3.20E-01 | 2.70E-02 | 4.90E-01 | 4.30E-  01 | 1.90E-02 |
|  | chr11:20408056 | DBX1 | 6.20E-02 | 9.20E-01 | 9.10E-01 | 4.60E-01 | 6.80E-01 | 9.30E-01 | 5.00E-01 | 9.80E-01 | 8.70E-01 | 4.80E-  01 | 6.20E-02 |
|  | chr11:20408056 | DBX1 | 7.70E-02 | 9.20E-01 | 7.40E-02 | 3.50E-01 | 2.40E-01 | 4.90E-01 | 5.70E-01 | 6.70E-01 | 4.40E-01 | 4.00E-  01 | 7.70E-02 |
|  | chr11:20408056 | DBX1 | 1.30E-01 | 8.50E-02 | 5.50E-01 | 8.10E-01 | 2.50E-01 | 1.80E-01 | 2.70E-01 | 4.80E-01 | 6.80E-01 | 1.50E-  01 | 1.30E-01 |
|  | chr11:20408056 | DBX1 | 3.60E-01 | 1.80E-01 | 7.30E-01 | 9.90E-01 | 3.40E-01 | 9.30E-01 | 4.40E-01 | 4.10E-01 | 2.00E-01 | 7.60E-  01 | 3.60E-01 |
| rs11025539 | chr11:20408056 | FLJ13439 | 6.10E-01 | 6.20E-01 | 3.90E-01 | 6.60E-01 | 2.20E-01 | 2.80E-01 | 6.70E-01 | 8.60E-01 | 1.50E-02 | 9.40E-  01 | 6.10E-01 |
|  | chr11:20408056 | FLJ13439 | 1.80E-01 | 5.10E-01 | 6.60E-02 | 8.30E-02 | 5.90E-01 | 7.90E-02 | 9.70E-01 | 5.30E-01 | 7.60E-02 | 6.60E-  01 | 1.80E-01 |
|  | chr11:20408056 | FLJ13439 | 8.30E-01 | 4.30E-01 | 7.60E-01 | 7.10E-01 | 2.10E-01 | 6.80E-01 | 2.70E-01 | 3.50E-01 | 9.20E-02 | 6.90E-  02 | 8.30E-01 |
|  | chr11:20408056 | FLJ13439 | 5.70E-01 | 6.90E-01 | 5.80E-01 | 3.00E-01 | 1.40E-01 | 4.60E-01 | 1.60E-01 | 3.70E-01 | 7.90E-01 | 1.70E-  01 | 5.70E-01 |
| rs11025540 | chr11:20408136 | DBX1 | 9.70E-02 | 1.80E-04 | 9.00E-02 | 6.70E-01 | 6.80E-01 | 1.40E-01 | 4.40E-01 | 9.10E-01 | 5.30E-01 | 8.20E-  01 | 8.50E-01 |
|  | chr11:20408136 | DBX1 | 7.00E-01 | 1.90E-02 | 5.90E-01 | 5.60E-01 | 8.90E-01 | 7.80E-01 | 5.40E-01 | 3.20E-01 | 2.70E-02 | 4.90E-  01 | 4.30E-01 |
|  | chr11:20408136 | DBX1 | 2.20E-01 | 6.20E-02 | 9.20E-01 | 9.10E-01 | 4.60E-01 | 6.80E-01 | 9.30E-01 | 5.00E-01 | 9.80E-01 | 8.70E-  01 | 4.80E-01 |
|  | chr11:20408136 | DBX1 | 2.20E-01 | 7.70E-02 | 9.20E-01 | 7.40E-02 | 3.50E-01 | 2.40E-01 | 4.90E-01 | 5.70E-01 | 6.70E-01 | 4.40E-  01 | 4.00E-01 |
|  | chr11:20408136 | DBX1 | 2.10E-01 | 1.30E-01 | 8.50E-02 | 5.50E-01 | 8.10E-01 | 2.50E-01 | 1.80E-01 | 2.70E-01 | 4.80E-01 | 6.80E-  01 | 1.50E-01 |
|  | chr11:20408136 | DBX1 | 9.00E-01 | 3.60E-01 | 1.80E-01 | 7.30E-01 | 9.90E-01 | 3.40E-01 | 9.30E-01 | 4.40E-01 | 4.10E-01 | 2.00E-  01 | 7.60E-01 |
|  | chr11:20408136 | FLJ13439 | 8.90E-01 | 6.10E-01 | 6.20E-01 | 3.90E-01 | 6.60E-01 | 2.20E-01 | 2.80E-01 | 6.70E-01 | 8.60E-01 | 1.50E-  02 | 9.40E-01 |
|  | chr11:20408136 | FLJ13439 | 8.20E-02 | 1.80E-01 | 5.10E-01 | 6.60E-02 | 8.20E-02 | 5.90E-01 | 7.90E-02 | 9.70E-01 | 5.30E-01 | 7.60E-  02 | 6.60E-01 |
|  | chr11:20408136 | FLJ13439 | 5.00E-01 | 8.30E-01 | 4.30E-01 | 7.60E-01 | 7.10E-01 | 2.10E-01 | 6.80E-01 | 2.70E-01 | 3.50E-01 | 9.20E-  02 | 6.90E-02 |
|  | chr11:20408136 | FLJ13439 | 5.50E-01 | 5.70E-01 | 6.90E-01 | 5.80E-01 | 3.00E-01 | 1.40E-01 | 4.60E-01 | 1.60E-01 | 3.70E-01 | 7.90E-  01 | 1.70E-01 |
|  | chr11:20408257 | DBX1 | 9.70E-02 | 1.80E-04 | 9.00E-02 | 6.70E-01 | 6.80E-01 | 1.40E-01 | 4.40E-01 | 9.10E-01 | 5.30E-01 | 8.20E-  01 | 8.50E-01 |
|  | chr11:20408257 | DBX1 | 7.00E-01 | 1.90E-02 | 5.90E-01 | 5.60E-01 | 8.90E-01 | 7.80E-01 | 5.40E-01 | 3.20E-01 | 2.70E-02 | 4.90E-  01 | 4.30E-01 |
| rs11025541 | chr11:20408257 | DBX1 | 2.20E-01 | 6.20E-02 | 9.20E-01 | 9.10E-01 | 4.60E-01 | 6.80E-01 | 9.30E-01 | 5.00E-01 | 9.80E-01 | 8.70E-  01 | 4.80E-01 |
|  | chr11:20408257 | DBX1 | 2.20E-01 | 7.70E-02 | 9.20E-01 | 7.40E-02 | 3.50E-01 | 2.40E-01 | 4.90E-01 | 5.70E-01 | 6.70E-01 | 4.40E-  01 | 4.00E-01 |
|  | chr11:20408257 | DBX1 | 2.10E-01 | 1.30E-01 | 8.50E-02 | 5.50E-01 | 8.10E-01 | 2.50E-01 | 1.80E-01 | 2.70E-01 | 4.80E-01 | 6.80E-  01 | 1.50E-01 |
|  | chr11:20408257 | DBX1 | 9.00E-01 | 3.60E-01 | 1.80E-01 | 7.30E-01 | 9.90E-01 | 3.40E-01 | 9.30E-01 | 4.40E-01 | 4.10E-01 | 2.00E-  01 | 7.60E-01 |
|  | chr11:20408257 | FLJ13439 | 8.90E-01 | 6.10E-01 | 6.20E-01 | 3.90E-01 | 6.60E-01 | 2.20E-01 | 2.80E-01 | 6.70E-01 | 8.60E-01 | 1.50E-  02 | 9.40E-01 |
|  | chr11:20408257 | FLJ13439 | 8.20E-02 | 1.80E-01 | 5.10E-01 | 6.60E-02 | 8.30E-02 | 5.90E-01 | 7.90E-02 | 9.70E-01 | 5.30E-01 | 7.60E-  02 | 6.60E-01 |
|  | chr11:20408257 | FLJ13439 | 5.00E-01 | 8.30E-01 | 4.30E-01 | 7.60E-01 | 7.10E-01 | 2.10E-01 | 6.80E-01 | 2.70E-01 | 3.50E-01 | 9.20E-  02 | 6.90E-02 |
|  | chr11:20408257 | FLJ13439 | 5.50E-01 | 5.70E-01 | 6.90E-01 | 5.80E-01 | 3.00E-01 | 1.40E-01 | 4.60E-01 | 1.60E-01 | 3.70E-01 | 7.90E-  01 | 1.70E-01 |

aveALL, average level of all brain regions; CRBL, cerebellum; FCTX, frontal cortex; HIPP, hippocampus; MEDU, medulla; OCTX, occipital cortex; PUTM, putamen; SNIG, substantia nigra; TCTX, temporal cortex; THAL, thalamus; WHMT, intralobular whiter matter.

Supplementary figure S1. Gene expression pattern of the five associated genes in human tissues in GTEX database. *GRM7*, *DOCK5*, and *SGCZ* were preferentially expressed in human brain (Figure a, b, and c; yellow box plot); *PRMT3* and *USP48* were highly expressed in human brain (Figure d and e; yellow box plot).


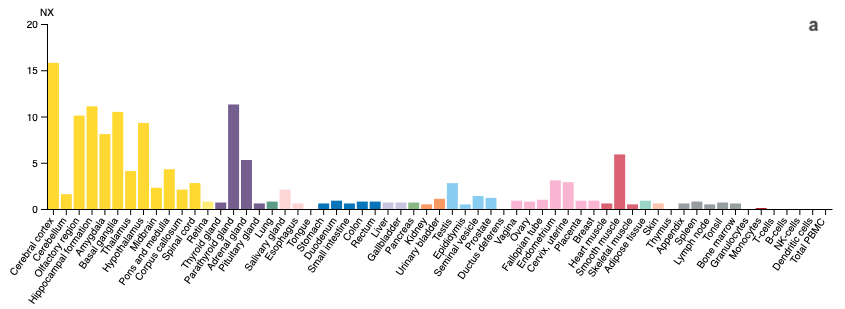


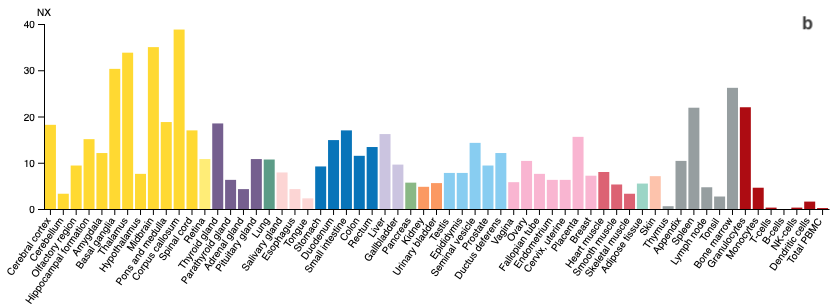


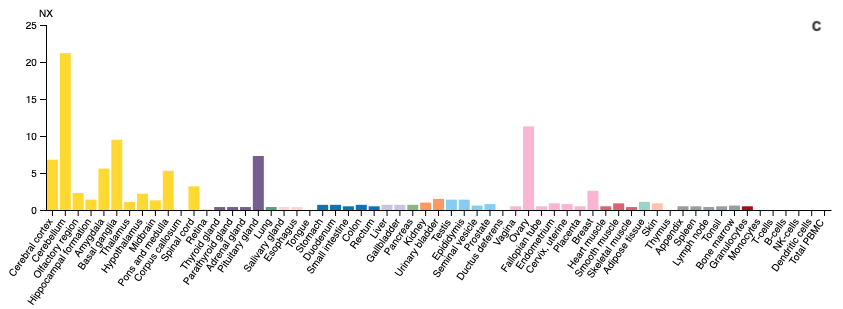


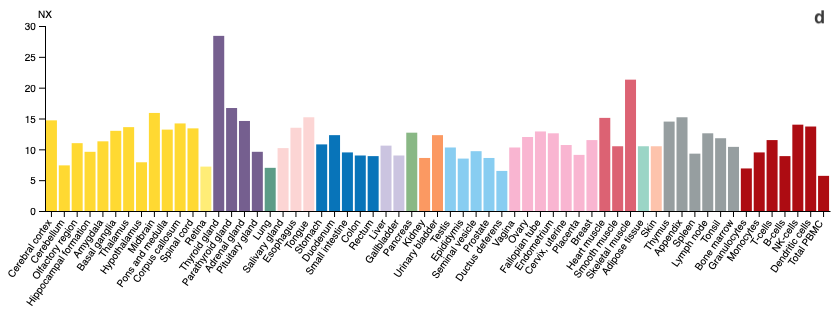


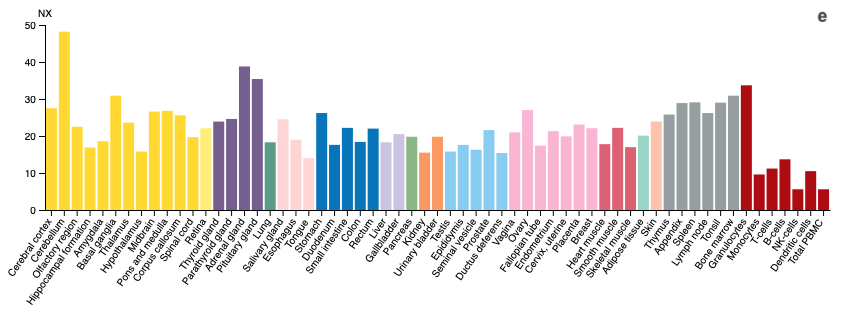

Supplement: Supplementary file 1 — Supplementary materials [file 41398_2022_1942_MOESM1_ESM.docx]
